# The rebound-initiating viral reservoir (RIVR) in SIV/SHIV infection is dominated by plasma virus sequences present at the time of ART initiation

**DOI:** 10.1128/jvi.00366-26

**Published:** 2026-07-28

**Authors:** Mykola Pinkevych, Steffen S. Docken, Yuhuang Wu, Timothy Schlub, Justin L. Harper, Elise G. Viox, Sadia Samer, Deanna Kulpa, Michael R. Betts, Brandon F. Keele, Mirko Paiardini, Katharine J. Bar, Deborah Cromer, Miles P. Davenport

**Affiliations:** 1 Infection Analytics Program, Kirby Institute for Infection and Immunity, UNSW Australia 7800 , Sydney, New South Wales, Australia; 2 Department of Mathematics, School of General Education, Jinhua University of Vocational Technology 686684, Jinhua, China; 3 Faculty of Medicine and Health, University of Sydney 522555, Sydney, New South Wales, Australia; 4 Division of Microbiology and Immunology, Emory National Primate Research Center, Emory University 1371, Atlanta, Georgia, USA; 5 Department of Pathology and Laboratory Medicine, School of Medicine, Emory University 12239, Atlanta, Georgia, USA; 6 Department of Microbiology and Center for AIDS Research, Perelman School of Medicine, University of Pennsylvania 14640, Philadelphia, Pennsylvania, USA; 7 AIDS and Cancer Virus Program, Frederick National Laboratory for Cancer Research, Frederick, Maryland, USA; 8 Department of Medicine and Center for AIDS Research, Perelman School of Medicine, University of Pennsylvania 14640, Philadelphia, Pennsylvania, USA; Icahn School of Medicine at Mount Sinai, New York, New York, USA

**Keywords:** SIV, HIV, latency, viral rebound, latent reservoir formation

## Abstract

Active human immunodeficiency virus (HIV) replication generates long-lived latently infected cells that persist despite effective antiretroviral therapy (ART). Several studies analyzing proviral DNA sequences sampled during ART have shown that these sequences are closely related to plasma virus present around treatment initiation. However, a large proportion of proviral DNA is defective or difficult to reactivate, suggesting that only a subset of latent proviruses contributes to viral rebound *in vivo*. Here, we used a barcoded simian immunodeficiency virus (SIV) or simian-human immunodeficiency virus (SHIV) model to study virus rebounding after antiretroviral treatment interruption (ATI) and its relationship to plasma virus present before ART initiation. We modeled barcode matches between rebound virus and pre-ART plasma virus to estimate the contribution of different time points to subsequent rebound. We estimate that plasma virus sampled immediately before ART contributes approximately 65%–95% of successful rebound lineages. This remains true after re-treatment and re-interruption of ART, with the circulating virus present just before the second ART contributing most to the second rebound. In contrast, analysis of proviral DNA during ART suggests that plasma virus present at the peak of primary infection contributes more substantially to the DNA reservoir (61%–90%), whereas the virus sampled immediately before ART contributes less. These findings suggest that the observable proviral DNA reservoir is established primarily during peak infection and is supplemented throughout untreated infection. However, post-treatment rebound virus originates from only a small subset of the total proviral DNA reservoir, predominantly formed around the time of ART initiation.

**IMPORTANCE:**

Human immunodeficiency virus infection cannot be eliminated because of long-lived, latently infected cells. Understanding when the latent reservoir forms may help identify the timing of interventions to reduce viral rebound. We analyzed data from macaques infected with a barcoded virus that allowed us to track individual viral lineages. Although many lineages were present in plasma during active infection, only a small number rebounded after antiretroviral treatment (ART) interruption. We compared barcode patterns in rebound virus with plasma virus sampled at different times before treatment to identify when rebounding lineages were observed. We found that the rebound virus was most closely related to the plasma virus present at treatment initiation, suggesting that this may be an important time for the formation of the rebound-initiating reservoir. Rebound virus matched pre-treatment plasma virus more closely than proviral DNA detected during ART, suggesting that on-treatment proviral DNA may poorly predict which viruses will re-emerge after treatment interruption

## INTRODUCTION

Although effective antiretroviral therapy (ART) can suppress the human immunodeficiency virus (HIV) in plasma below the limit of detection, viral replication typically rebounds within weeks of treatment interruption due to the presence of a long-lived reservoir of infected cells. Investigation suggests that a pool of long-lived infected cells is established extremely early in the course of infection (1–3). This pool of long-lived infected cells is maintained and added to during active infection and persists during ART through a combination of cellular survival and clonal expansion. Analysis of proviral sequences suggests that much of this reservoir in HIV is not replication-competent due to large deletions, frameshifts, or hypermutation of the provirus (4, 5). Even if the provirus is intact and replication-competent, it may be only rarely reactivated, either because of epigenetic silencing of the proviral genome or integration into inactive sites of the host genome (6–8). A variety of assays have been developed to test the reactivation competence of the provirus by either sequencing or by *ex vivo* stimulation of cells (9–15). Analysis of the dynamics of HIV rebound after treatment interruption suggests that HIV successfully rebounds around once a week and that rebound is typically initiated by a small number of viral clonotypes (16). However, identifying the origins of the plasma virus sequences seen during rebound among the pretreatment plasma virus and on-treatment proviral sequences can be challenging (17–19).

A variety of different terms and definitions have been used to describe a range of long-lived HIV-infected cells (4). For example, the “total proviral DNA” might refer to any cell with provirus detected *in vivo*. However, this population includes cells infected with defective viral genomes, which, although they may be able to generate RNA or some protein, are not able to reignite virus replication after ART interruption. The reservoir can further be defined by having an intact proviral genome, but this likely overestimates the proportion of provirus that can truly reactivate to replicate systemically. Different definitions of the “replication-competent” or “rebound-competent” viral reservoirs aim to identify the subset of cells with proviruses capable of successful replication. One means of identifying cells capable of replication is through *ex vivo* stimulation of cells. However, the number of cells that can be induced *ex vivo* to generate virus replication is an underestimation of the true reactivation-competent reservoir, as *ex vivo* reactivation is stochastic and no method of cell stimulation is fully effective (4, 9–13). The major obstacle to long-term remission following treatment interruption in HIV is the subset of infected cells that successfully initiates the rebound of plasma viremia. This is best identified as the subset of the viral population that can be observed replicating in plasma after treatment interruption. We refer to this as the “rebound-initiating viral reservoir” (RIVR) to differentiate this from other descriptions of the HIV reservoir (such as the rebound-competent viral reservoir, estimated by quantitative viral outgrowth assay [QVOA], of which only a small subset actually reactivates to form the RIVR [20, 21]).

It is well established that a rebound-initiating viral reservoir is formed at the early stage of HIV/SIV infection, since post-treatment rebound can be observed even after early anti-retroviral treatment initiation (at a stage when there are only very low levels of provirus detectable) (3, 22). However, the genetic analysis of the DNA reservoir in people living with HIV (PLWH) who initiated ART in chronic infection reveals that despite the presence of the proviral DNA from many stages of infection, the majority of proviral DNA in the latent pool is closely related to the viral variants that were present in plasma just before treatment initiation (23–27). Similarly, studies of the viral variants induced in a QVOA *ex vivo* found that the highest proportion of outgrowth variants was seeded around the time of ART initiation (23, 28–30). The limited studies of the origins of rebound virus after treatment interruption also suggest that much of this virus originates around the time of anti-retroviral treatment initiation (Table S9.1) (27, 31). This is consistent with studies demonstrating a rapid turnover of infected cells during active replication, leading to the elimination of older variants (26, 32, 33). Combined, these studies suggest that at least some aspects of the HIV/SIV reservoir are relatively labile during untreated infection and that replicating virus present at the time of initiation of antiretroviral therapy may make a disproportionate contribution to the reservoir. Thus, the time of antiretroviral initiation may represent an important window for reservoir formation and a major opportunity to limit reservoir establishment (34, 35).

Although several studies have analyzed the temporal origins of HIV provirus and inducible virus, identifying the timing of when the RIVR is formed is challenging in HIV. This is due to the need for single-genome analysis to identify individual clonotypes (limiting the total number of clonotypes that can be identified). In addition, identification of viral origins in such studies typically relies on phylogenetic analysis to look at the similarity of rebounding and proviral sequences (23–31). Finally, variability in the timing of ART initiation and timing of both pre-ART plasma virus and rebound plasma virus creates an additional obstacle to determining the timing of the relationships between rebounding virus and earlier plasma or proviral sequences. The use of barcoded SIV/SHIV overcomes many of these limitations. It allows deep sequencing to better characterize virus populations and to uniquely identify (barcode) lineages across different time points (36, 37). In the current study, we use data from rhesus macaques (RMs) infected with barcoded SIV or SHIV that underwent ART initiation at different times following infection. By comparing the barcodes that rebounded after treatment interruption with barcodes observed in the plasma virus at different sampling times before ART and in proviral DNA prior to ART, we can study the temporal origins of the rebound-initiating viral reservoir.

## RESULTS

### Infection with barcoded SIV/SHIV

**Fig 1:**
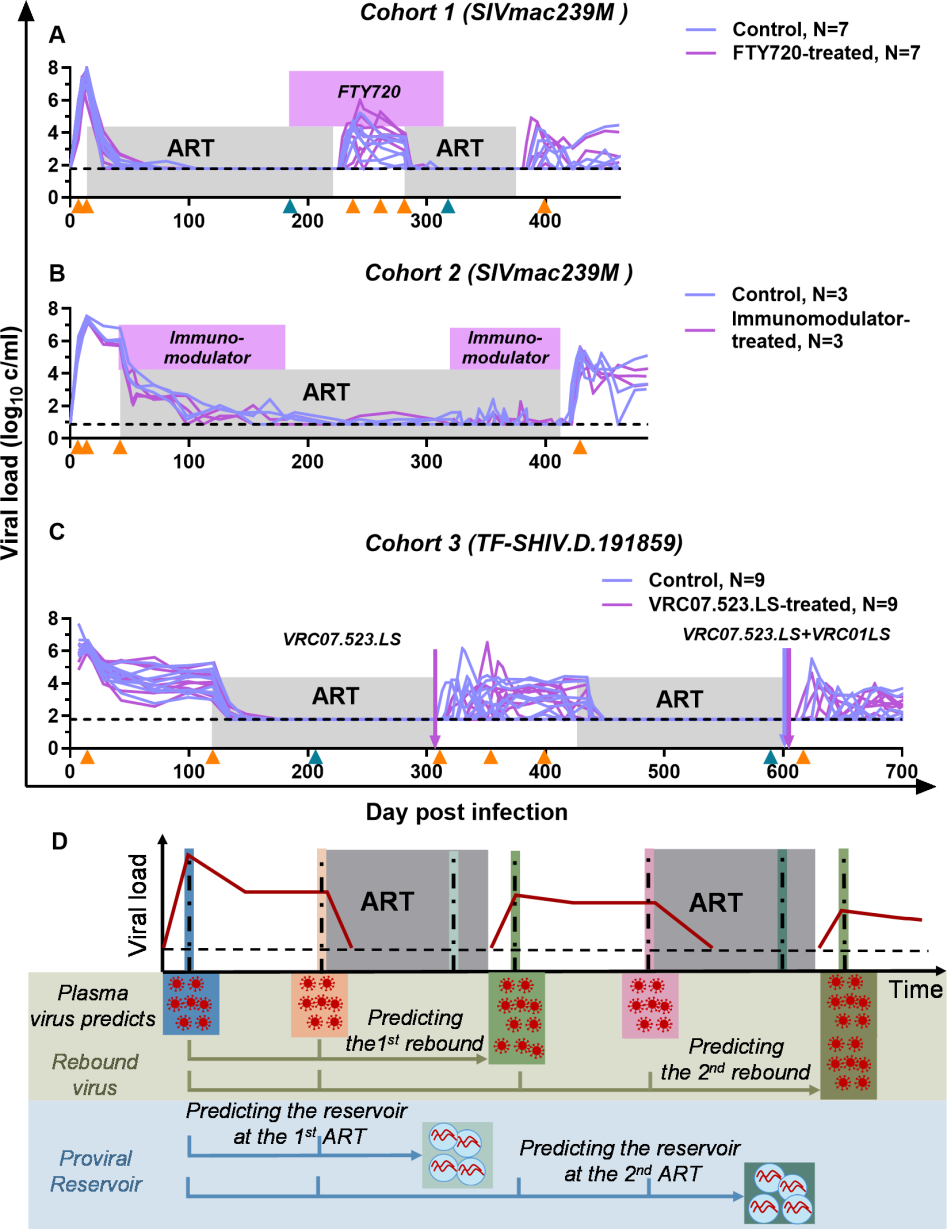
The ART initiation, interruption, and intervention timing in cohorts of macaques used in the current study and the study design. Gray shaded areas are the time intervals of ART. (**A**) Seven macaques in Cohort 1 were treated with FTY720 during the time interval shown as a purple bar. (**B**) Three macaques in Cohort 2 underwent three treatment cycles with an experimental immunomodulator during the interval shown in the panel by purple bars. (**C**) Nine macaques from Cohort 3 received VRC07.523LS bNAb at the beginning of the first ART interruption (purple arrow), and then all animals were treated with VRC07.523LS and VRC01LS at the start of the second ART interruption, as shown by blue and purple arrows. Orange and blue triangles represent median sampling times (since subjects were sampled at different times) for barcodes in plasma. (**D**) Schematic representation of the design of the current study. Using plasma viral loads for different barcodes at different time points before ART initiation, we can predict rebound at the first treatment interruption and proviral DNA before it. By adding barcodes from the first treatment interruption to those from pre-treatment, we can predict rebound at the second treatment interruption and proviral DNA at the second ART.

In this study, we use samples from two longitudinal cohorts of RMs infected with barcoded SIVmac239M (36) and one cohort of RM infected with barcoded SHIV.D.191859 (SHIV.D) (38) (Fig. 1). In the first cohort (39), 14 animals were infected with SIVmac239M and initiated ART with emtricitabine (FTC), tenofovir (TDF), and dolutegravir (DTG) at 14 days post-infection (p.i.) and were maintained on ART for 205 or 206 days. Half of the animals were treated with FTY-720 (fingolimod) just before anti-retroviral treatment interruption (ATI). All animals rebounded during ART interruption, and ART was reinitiated 61 or 63 days after the first ART interruption. A second anti-retroviral treatment interruption occurred after 93 days of the second antiretroviral treatment cycle. Barcode sequencing of plasma virus from four time points was compared to provirus from peripheral blood mononuclear cells (PBMCs) and lymph node mononuclear cells (LNMCs) from two time points during the two treatment interruptions.

In the second cohort, six animals were infected with SIVmac239M and initiated treatment with FTC, DTF, and DTG at 42 days post-infection. Three animals were treated with an experimental immunomodulator at ART initiation and before ATI (see Materials and Methods and Table S1.1 for the protocol). ART was continued for 370 days before anti-retroviral treatment interruption. Three plasma virus time points before ART initiation were available for comparison with the rebound virus.

In the third cohort (38), 18 animals were infected with SHIV.D and commenced antiretroviral treatment at 120 or 121 days post-infection. Treatment was maintained for 178 or 194 days before the first antiretroviral treatment interruption. Half of the animals were treated with VRC07.523LS during the first treatment interruption. ART was re-initiated after a 120- or 121-day treatment interruption and continued for 161–189 days before a second antiretroviral treatment interruption. All animals were treated with VRC07.523LS and VRC01LS immediately prior to the second ATI. Barcode sequences from plasma samples were available for four time points, and PBMC proviral sequences from samples for two time points for comparison with the rebound virus.

### Using barcodes to infer the origins of the rebounding virus

To determine the time of seeding of the rebounding virus, we compared the viral barcodes that were detected in plasma following ATI with those from plasma virus sampled at different times pre-ART. We developed a mathematical model that tests how well the barcode frequency in plasma virus at different times pre-ART predicts the barcodes detected after anti-retroviral treatment interruption.

**Fig 2:**
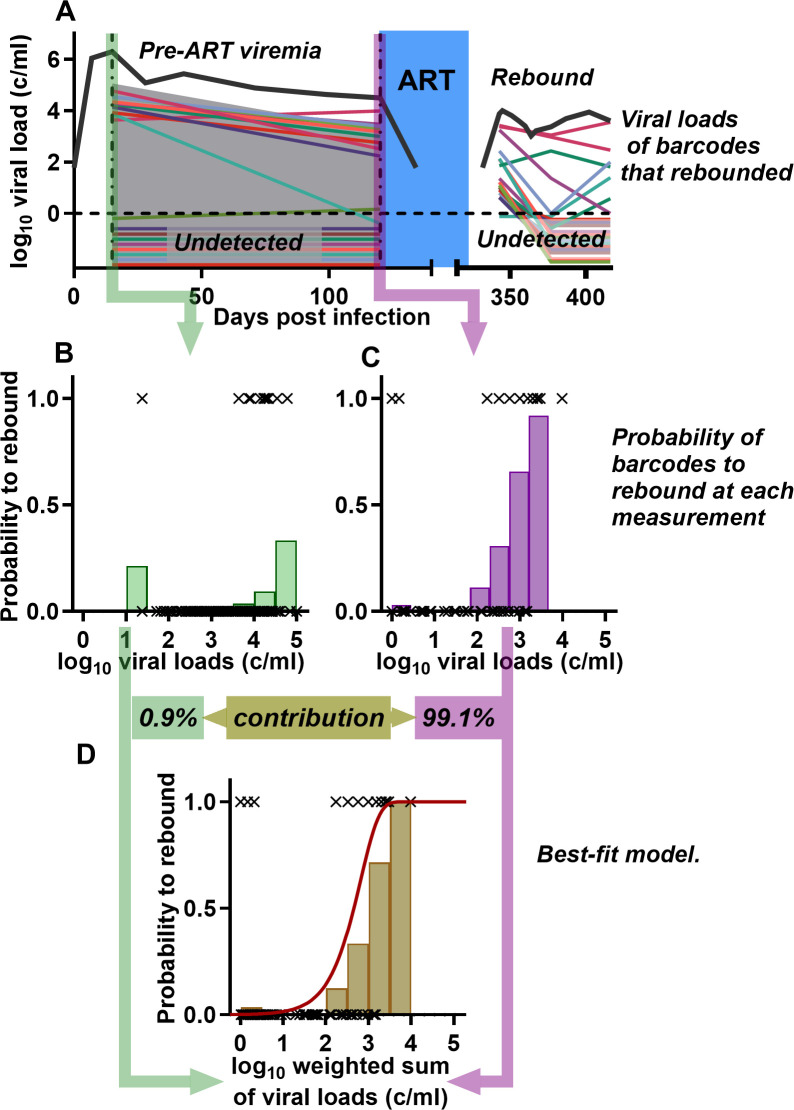
Modeling to identify the source of viral rebound. (**A**) Initial data and the best-fit model using subject 18_RBm17 from Cohort 3 as an example. The total viral load is a thick black line; the viral load of each barcode that rebounded at treatment interruption is a thin colored line. The span between the maximal and minimal viral load of barcodes that did not rebound is indicated by the gray shaded area. Timing of plasma virus sampling and sequencing is indicated by vertical dash-dotted lines and green and purple arrows. (**B and C**) The frequency of detection (probability to rebound) of barcodes at ART interruption (y-axis value of the bars) with the pre-ART viral load falling in the viral load range indicated by the width of the bars. Black X indicates the plasma viral loads of non-rebounded (0 on the y-axis) or rebounded (1 on the y-axis) barcodes. For barcodes present in plasma at day 15 (**B**), viral loads observed at both high and low copy numbers are seen in the rebound viraemia (i.e*.,* plasma virus copy number at day 15 is a poor predictor of rebound). For barcodes present in plasma at day 120 (**C**), the copy number of barcodes is a good predictor of the probability of rebound. (**D**). The best-fit model to predict rebound. The x-axis is the sum of fractions of viral loads (weighted sum) from plasma reads on days 15 and 120 according to the best-fit model. Symbol X denotes the weighted sum of viral loads for individual barcodes presented in panels B and C. The model predicts that day 15 contributes 0.9% and day 120 contributes 99.1% to the probability of a barcode being observed during viral rebound. The dark red line is the best-fit probability of rebound given the contribution to the virus from different time points.

By fitting the model to the data, we can estimate the fraction of proviral DNA contributed by each time point before ART that maximizes the likelihood of observing the set of barcodes detected after ART interruption (Fig. 2A through D).

For example, a specific time point in an animal would have a higher contribution to RIVR if the most abundant barcodes in plasma at that time point were the same barcodes that were observed to rebound after ART interruption (Fig. 2C). In contrast, if there were no relationship between plasma viral loads of barcodes at a particular time point and whether subsequently rebounded, then this time point would score as a lower contributor to RIVR (Fig. 2B).

We should note that the conclusion of the model depends only on the relative viral load in plasma in different measurements; the result does not depend on initial viral load and the number of barcodes in plasma before and after ART. However, a greater number of barcodes can make the fitting results more reliable.

Due to variable viral load that affects the abundance of barcodes (Fig. 2A), we will report the contribution of the plasma virus to the reactivation initiating reservoir in two ways: (i) the total contribution of the plasma virus at that time point to the later reactivation or (ii) the normalized contribution from the plasma virus at that time point (e.g., asking “what is the contribution made by a single copy of plasma virus at a given time point to the later reactivation observed?”). The latter analysis accounts for the different viral loads at different times to assess the effective contribution “per plasma RNA copy.” The mathematical models are detailed in the methods section.

### The contribution of plasma virus from different time points to the rebound-initiating viral reservoir

**Fig 3:**
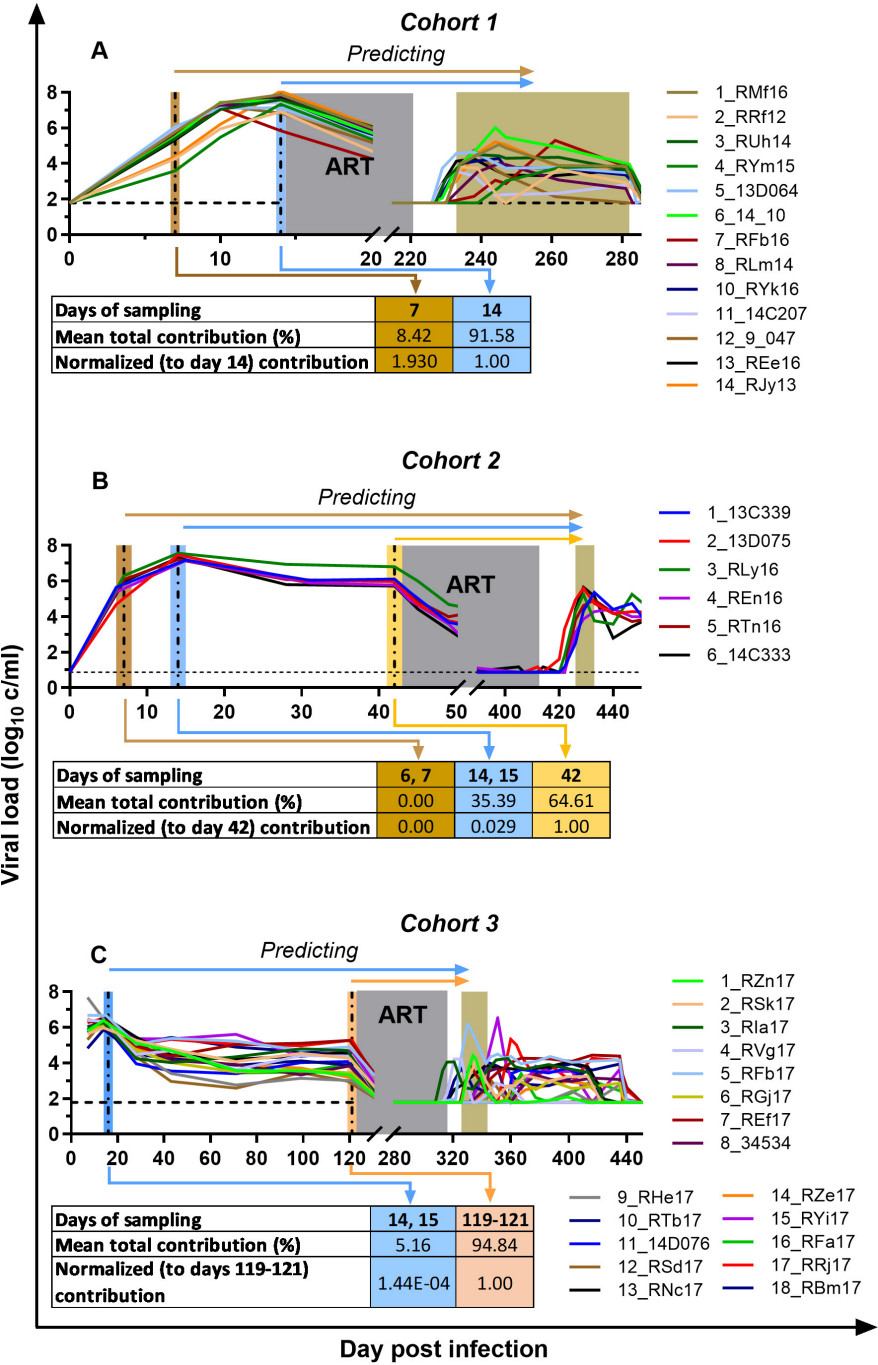
Modeling the contribution of plasma virus barcode to the post-treatment rebound virus. The trajectory of plasma viral load for individual animals is shown for macaques in Cohort 1 (**A**), Cohort 2 (**B**), and Cohort 3 (**C**). Vertical dash-dotted lines with color shade indicate the sequencing time points for subjects in the cohort. Khaki-shaded areas are sequencing times after rebound (different for individual macaques depending on the timing of rebound). The tables below these graphs show the estimated contributions of plasma virus at the different sequencing times to the observed rebound barcodes. Both the mean total contribution from plasma virus, as well as the normalized (“per-plasma-virion”) contribution, are shown for the different time points. The modeling shows that the major contribution to the rebound-initiating viral reservoir and the normalized contribution arise from close to the time of ART initiation, independent of the timing of the treatment. The best-fit parameters and the percent total contribution for individual animals from each cohort are in Tables S2.1 to S2.3 for Cohorts 1, 2, and 3, respectively. The respective graphs of the best-fit functions are in Fig. S2.1 to S2.3.

We analyzed data from three independent cohorts of RMs to determine the total and normalized contribution of plasma virus at different time points to the rebound-initiating viral reservoir. In the first cohort, RMs (*N* = 13) were infected with SIVmac239M (36), treated with ART on day 14 after infection, and antiretroviral treatment was interrupted on days 219 or 220. The details and results of this study have been previously published (39). Pretreatment sequencing of plasma was conducted on days 7 and 14 post-infection. Post-rebound plasma virus was sequenced 2–4 times between days 234 and 281 post-infection (depending on the timing of rebound in individual animals). Our model suggests that overall, the virus present in the plasma on day 14 contributed on average >91% (SD 16.6%) of the rebound-initiating viral reservoir. However, given that there was 118-fold more virus present on day 14 than on day 7, it is perhaps not surprising that virus present on day 14 made such a large contribution. To account for this, we can also estimate the normalized contribution to the rebound-initiating reservoir of a single plasma virus copy (or a single copy of a barcode) present in the plasma on days 7 or 14. We call this relative contribution the “normalized” contribution. We find that once the viral load at each sampling day is accounted for, there is an almost 2-fold higher normalized “per plasma virion” contribution from virus present on day 7 than day 14 (Fig. 3A) (i.e*.,* for every copy of a virus observed in the plasma virus on day 7, it had about twice the chance of being observed in the rebound virus population compared to a copy of plasma virus at day 14). A major caveat in this cohort is that the two samples were taken very close together (hence, the barcode frequencies were very similar) and that treatment was initiated at the peak of viremia.

A second cohort of RMs (*N* = 6) was infected with the same barcoded SIVmac239M virus stock and initiated ART on day 42. Treatment was continued until day 412. Plasma virus was sequenced on days 6 or 7, 14 or 15, and 42 after infection. We found that the best-fit parameters of our model suggest that plasma virus observed on the day of treatment (day 42) makes a greater contribution to the rebound-initiating reservoir than virus present at the peak of infection (days 14 or 15), with a mean of 64.6% (SD 14.3%) vs. a mean of 35.4% (SD 14.3%), respectively, despite there being a 20-fold higher viral load at days 14 or 15 than there was at day 42 (Fig. 3B). Indeed, when we consider the normalized contribution to the reactivation-initiating reservoir of the virus present on the day of treatment, we find that barcodes sampled in plasma at the time of ART initiation make a 30-fold higher normalized contribution to the rebounding virus compared to the barcodes present at the peak of infection. The estimated normalized contribution from the plasma virus barcodes early in infection (days 6 or 7) is equal to 0.

A third cohort of animals (*N* = 18) was infected with a barcoded SHIV.D.191859 (38). Antiretroviral therapy was initiated on days 120 or 121 and continued for 178 or 194 days. Plasma virus was sequenced on days 14 or 15 and on days 119–121 post-infection (the latter immediately prior to ART initiation). In this cohort, we estimate that plasma virus barcodes observed at the time of ART initiation contribute 94.8% (SD 7.8%) to the rebound-initiating SHIV reservoir, while virus present at the peak of primary infection accounted for only 5.2% (SD 7.8%) of the observed rebounding virus. The normalized contribution of virus present at the time of treatment to the rebound-initiating viral reservoir is almost 7,000 times greater than that of virus present at the peak (Fig. 3C).

### Understanding viral rebound after reinitiation of ART and a second treatment interruption

The analysis above estimates the contribution of plasma virus at different times before ART initiation to the rebound-initiating viral reservoir. However, animals from Cohorts 1 and 3 recommenced ART after the first interruption and subsequently underwent a second ART treatment for 3 and 6 months, respectively, before undergoing a second ATI. This allowed us to examine the rebounding plasma virus barcodes observed in the second interruption and analyze the relationship to the plasma virus sampled at different time points, both before the first ART initiation and the plasma virus circulating during the first antiretroviral treatment interruption. For this analysis, we required animals to have plasma virus barcodes sequenced from both the first treatment interruption (both early and late) and during the second treatment interruption. This reduced the available animals to *n* = 7 for the first cohort and *n* = 12 for the third cohort.

**Fig 4:**
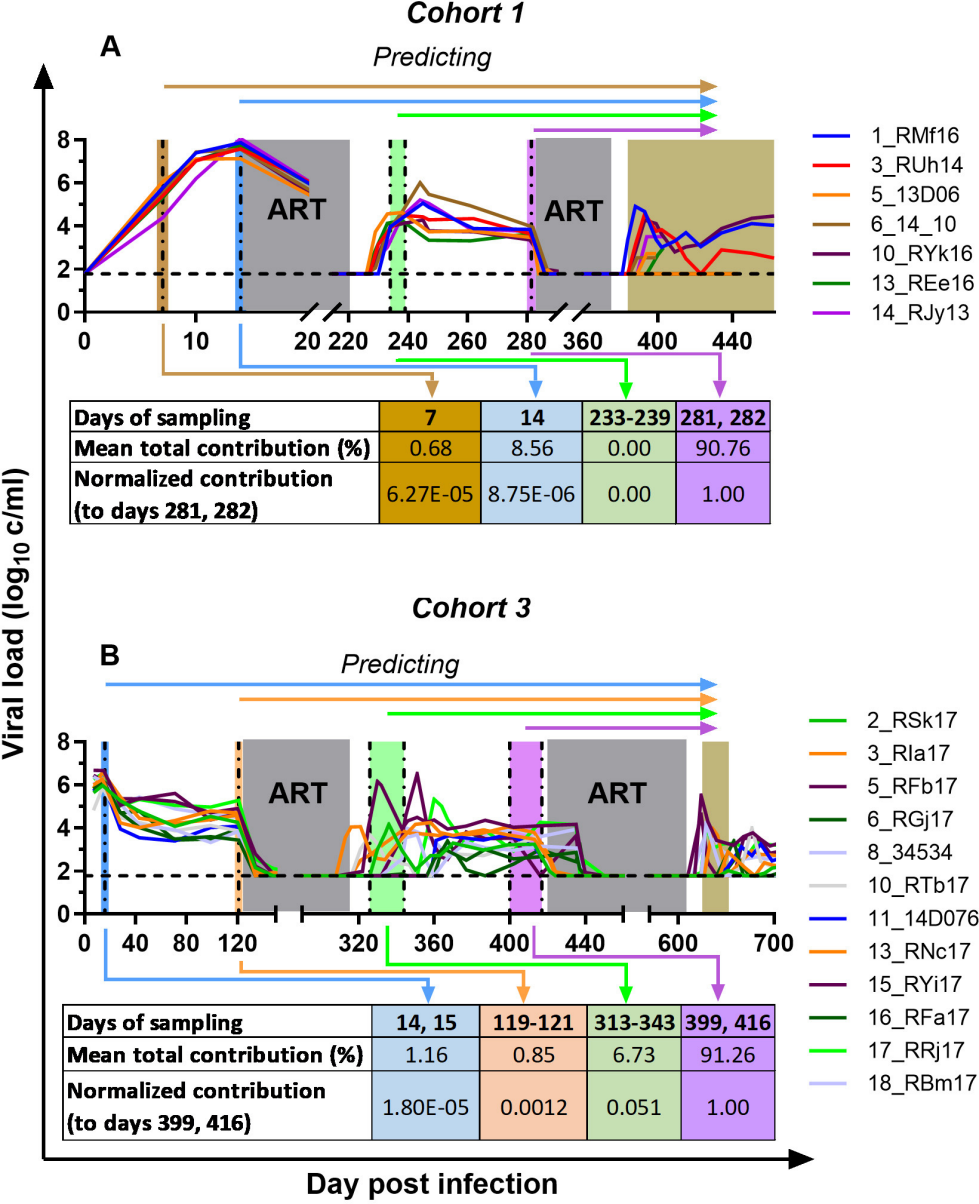
Modeling the contribution of plasma virus at different time points to the observed rebound at the second treatment interruption. The trajectory of viral load for individual macaques of Cohort 1 (**A**) and Cohort 3 (**B**) is shown across two ART treatment cycles. The colored shaded area between dash-dotted lines is the time interval when sequencing was conducted (which differed between individual animals). Khaki-shaded areas indicate sequencing times after rebound. The tables and graphs below indicate the estimated contributions of plasma virus barcodes at different time points to the observed rebound at the second treatment interruption. The best-fit parameters and the percent total contribution for individual animals are shown in Tables S3.1 and S3.2 for Cohorts 1 and 3, respectively. The respective graphs of the best-fit functions are shown in Fig. S3.1 and S3.2.

We fitted a model (equation 2) that included data from all of the available plasma virus samples (from both pre-ART and the first ART interruption) as predictors of barcodes present in the rebound virus observed during the second antiretroviral treatment interruption. For both cohorts, we found that the time point immediately prior to the second ART initiation contributed the most to the rebounding barcodes observed after the second treatment interruption (Fig. 4) (90.8% [SD 4.1%] and 91.3% [SD 9.6%] for Cohorts 1 and 3, respectively). This is despite the fact that the viral load observed just prior to the second ART initiation is >10,000-fold and >500-fold, respectively, lower than the viral load observed at the peak of primary infection. The importance of the last time point prior to the second treatment initiation is even greater when we consider the normalized contribution of a single virion present at each time point to the rebound-initiating viral reservoir (Fig. 4).

### Timing of seeding the total proviral DNA reservoir in PBMCs and LNMCs

The analysis above suggests that plasma virus replicating at ART initiation contributes a very large proportion of the rebound virus seen during subsequent ART interruption and that each new ART initiation seems to “reset the clock” (i.e., the viruses replicating immediately before the most recent ART initiation are most likely to rebound at the subsequent ATI). The origin of rebound in this study contrasts with previous studies of the temporal origin of the reservoir (assessed via either sequencing of the proviral DNA reservoir or of virus produced after *ex vivo* stimulation (QVOA)), which suggested that only around half of these (pro-)viral populations originate around ART initiation. Therefore, we estimated the contribution of plasma virus at different time points off-ART to the SIV/SHIV proviral DNA in PBMCs and LNMCs sampled during ART. We applied our model (equation 2) to assess the contribution of plasma virus at different time points to the barcodes detected via direct sequencing in PBMC or LNMC during ART in Cohorts 1 and 3, where proviral sequences were available.

**Fig 5:**
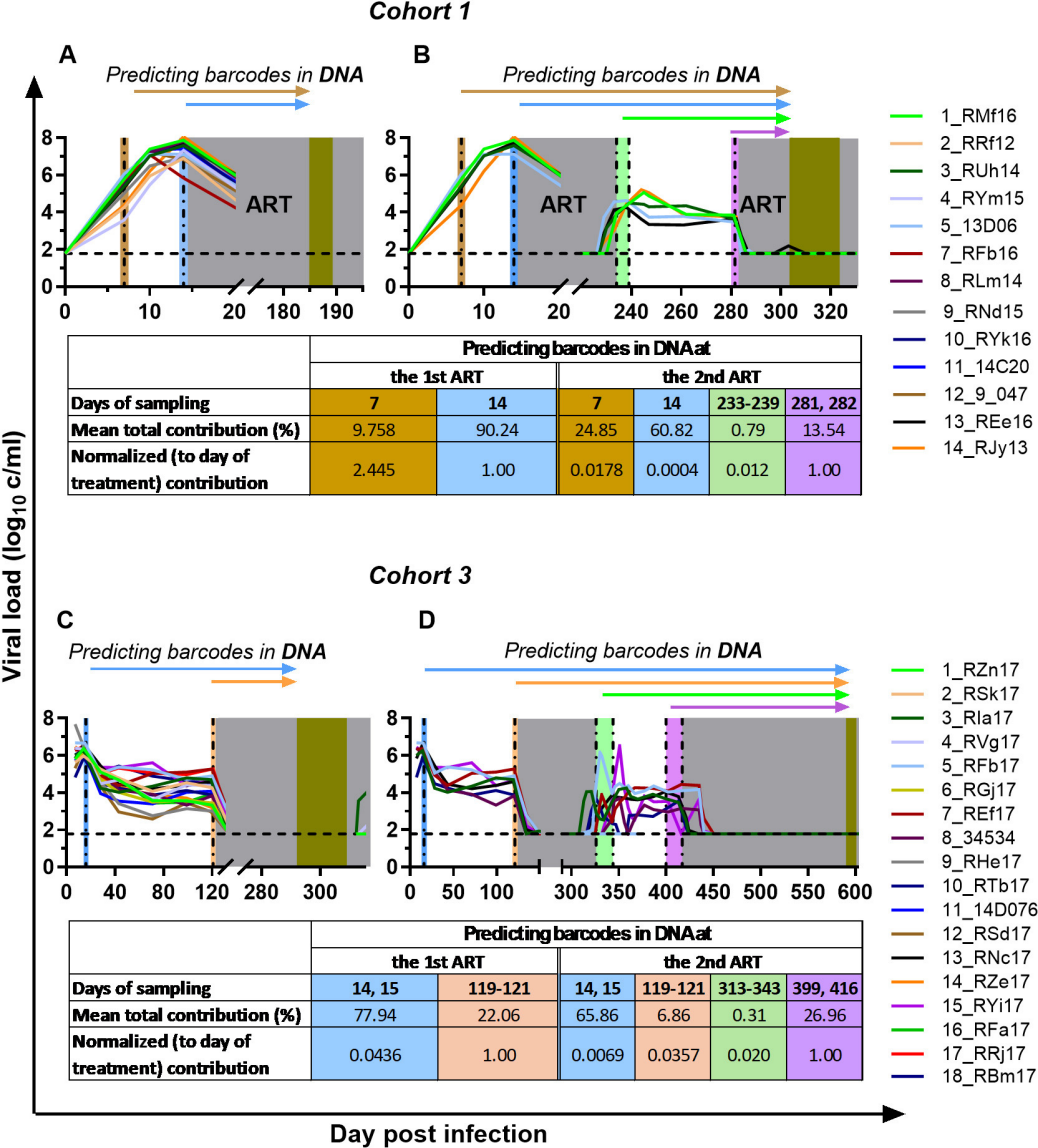
Modeling the contribution of plasma virus to the proviral DNA in PBMCs or lymph nodes during the first and the second ART. The dynamics of plasma viral load and the time of plasma and proviral sampling are shown for individual animals from Cohort 1 (**A and B**) and Cohort 3 (**C and D**). The estimated total contribution to proviral DNA and normalized contribution for different time points are indicated in the tables below. For both cohorts, the plasma virus at the peak on days 14 and 15 is the major contributor to proviral DNA (predicting the appearance of a barcode in either PBMCs or lymph nodes) at the first (**A and C**) and the second (**B and D**) ART, although the plasma virus at ART initiation still makes the highest normalized (per plasma virion) contribution. The best-fit parameters and the percent total contribution for each subject are in Tables S5.1 to S5.4. The respective graphs of the best-fit functions are in Fig. S5.1 to S5.4.

The analysis revealed very different temporal origins of the total proviral DNA reservoir on ART compared with the plasma rebound virus RNA after treatment interruption. In animals treated on day 14 after infection (Cohort 1), around 90% (SD 18.3%) of the total proviral DNA during ART originated from the peak of infection (which was also the time of ART initiation). However, for animals treated around day 120 following infection (Cohort 3), plasma virus sampled at ART initiation contributed only 22.1% to the total proviral DNA observed on ART, and 77.9% of the proviral DNA originated from the peak of infection on day 14 (Fig. 5A and C).

We used the same approach to estimate contributors to proviral DNA seen during the second round of ART. In this case, we estimated that plasma virus observed just prior to the second ART initiation contributed 13.5% (SD 3.3%) in Cohort 1 and 26.0% (SD 12.4%) in Cohort 3 to the proviral sequences in the second ART phase. The plasma virus at the peak of primary infection remained the major contributor to the proviral pool, contributing 60.8% in Cohort 1 and 65.9% in Cohort 3 (Fig. 5B and D). Thus, in both animal cohorts, the majority of the total proviral DNA reservoir assessed over more than a year of the study was seeded at peak viremia, regardless of whether ART was initiated during acute (day 14) or relatively early (day 120) infection, and regardless of a subsequent ATI with weeks of virus replication.

### Predicting rebounding barcodes from proviral DNA

We also examined whether sequences present in the proviral DNA during ART are able to predict the rebounding virus and, in particular, to compare plasma virus (prior to ART) versus provirus (on ART) as predictors of barcodes observed in plasma after subsequent treatment interruption. In contrast to plasma virus, where we can measure the total copy number per milliliter of plasma for different barcodes, for proviral DNA, we are only able to determine the proportion each barcode contributes to the total proviral DNA (measured in PBMCs or LNMCs). Therefore, we applied our model (equation 2) to plasma virus and proviral DNA separately, using a model comparison approach (Akaike information criterion [AIC]) to check the goodness of fit (Table S6.1). We found that models incorporating the plasma virus provided a consistently better fit (by AIC) than those using proviral DNA (Table S6.1). This is consistent with the results above, where plasma virus immediately prior to ART is a good predictor of rebound, but these sequences are poorly represented in proviral DNA prior to treatment interruption. However, we should note that the number of barcodes detected in proviral DNA is usually lower than that in plasma before ART initiation, which can lower the reliability of fitting results when using proviral DNA as a predictor.

### Plasma virus levels prior to ART initiation predict the frequency of viral rebound

**Fig 6:**
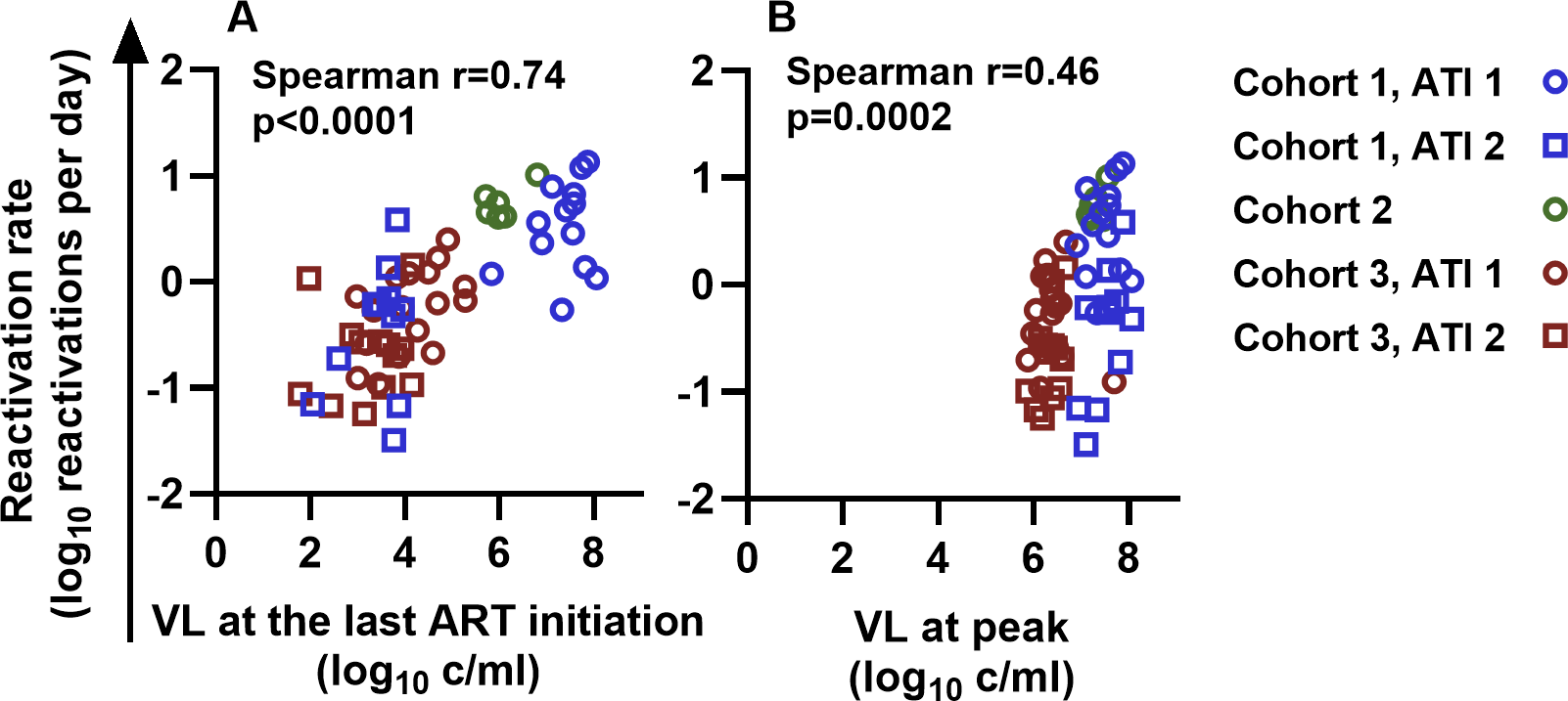
The viral load at ART initiation is a better predictor of the reactivation rate than the viral load at peak. (**A**) Reactivation rate at the first and the second ATIs vs. viral load at treatment before the first and the second ART, respectively, Spearman r = 0.74 (*P* < 0.0001). (**B**) Reactivation rate at the first and the second ATIs vs. viral load at pre-treatment peak, Spearman r = 0.46 (*P* = 0.0002). The method of estimation of the reactivation rate is published in a previous study (40).

Our analysis suggests that rebound virus clonotypes are closely related to the clonotypes present just before ART initiation and therefore that the rebound-initiating viral reservoir is formed at this time. One obvious prediction is that the frequency of viral rebound after ART interruption might also be determined by the plasma virus levels at the time of ART initiation. To examine this, we estimated the frequency of viral rebound (i.e., how many clonotypes rebound per day) using a method previously described (36, 40). We then compared the correlation between plasma viral load at peak, plasma viral load at ART, and rebound frequency. Both viral load at treatment initiation and viral load at peak are significantly correlated with the reactivation rate after treatment interruption (and are also significantly correlated with each other, Spearman r = 0.34, *P* < 0.0036). The correlation with viral load at treatment initiation (Spearman r = 0.74, *P* < 0.0001) is stronger than the correlation with viral load at peak (r = 0.46, *P* = 0.0002). Thus, viral load at treatment may also be a better predictor of the frequency of SIV/SHIV reactivation after ART interruption (Fig. 6).

## DISCUSSION

A number of studies have previously investigated the timing of the establishment of the HIV reservoir. These have mainly focused on the analysis of proviral DNA from PBMCs on ART treatment (23–27, 29, 31, 32, 41–43) or on sequences detected in *ex vivo*-reactivated virus induced via QVOA (23, 28–30). The actual viruses that rebound and replicate systemically *in vivo* after treatment interruption have only been studied in a few cases (27, 31). These studies have used phylogenetic comparisons of viral sequences to infer the origins of the observed (pro-)virus. Such studies have inferred that most of the proviral DNA sequences observed during ART (42%–60%) can be sourced to plasma virus replicating close to the time of ART initiation (median among the studies 0–1 years) (23–26) (Table S8.1). Studies of *ex vivo*-reactivated virus suggest that a higher fraction of sequences (49%–89%) may originate around the time of ART initiation (23, 25–31). In the small number of studies looking at rebound plasma virus (*n* = 2 patients, with *n* = 5 and 56 rebound sequences), they estimated that around 75%–80% of sequences came from around ART initiation (27, 31).

**Fig 7:**
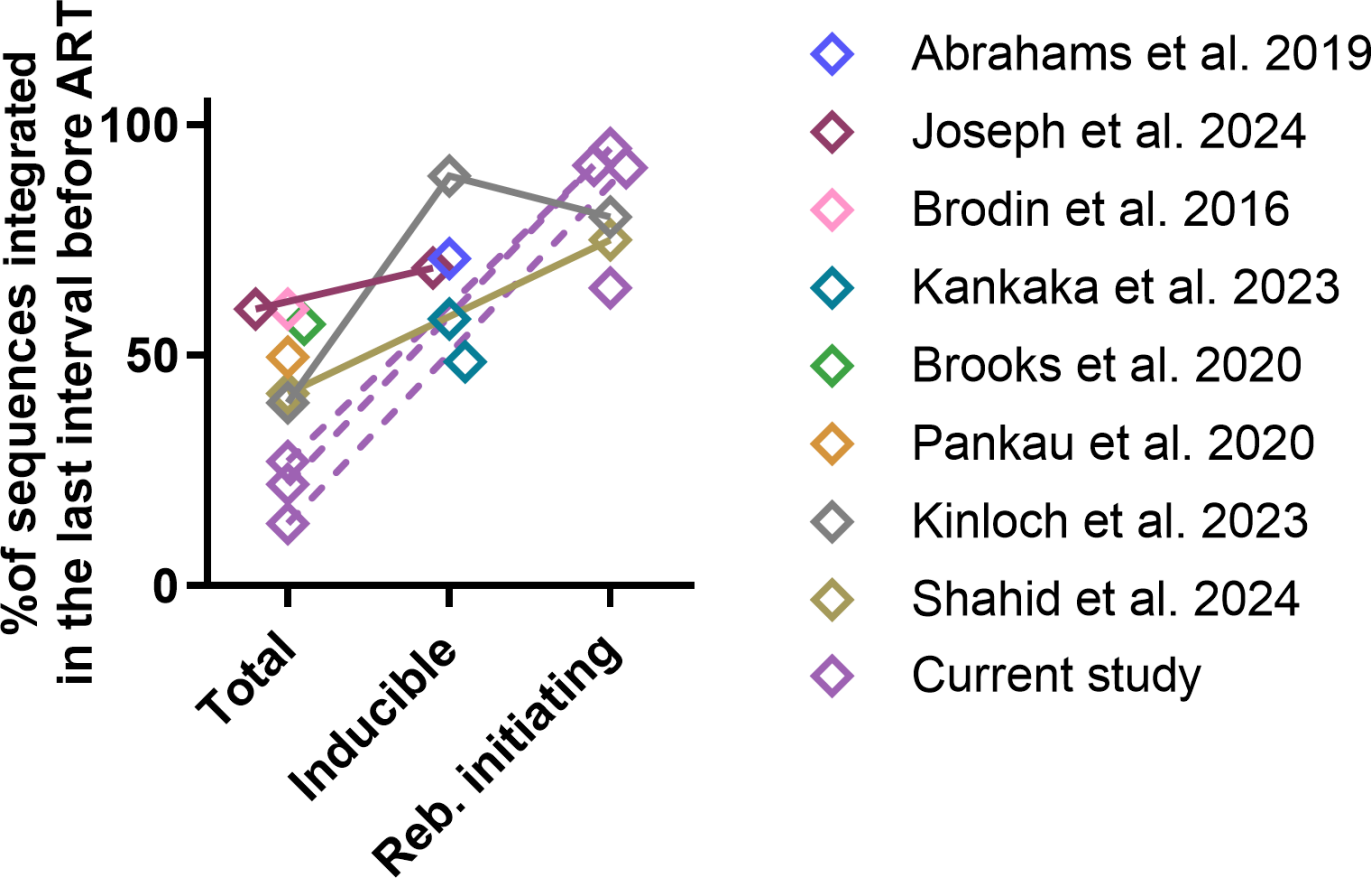
Comparison of the contribution of plasma virus to different measures of HIV or SIV/SHIV reservoir. This figure aggregates data from this study (purple) and published studies of the origins of the SIV/SHIV/HIV reservoir. Studies have measured either total proviral DNA, *in vitro*-reactivated virus, or rebounding virus (x-axis). The estimated contribution of plasma virus from the most recent pre-ART sample was extracted from the published studies. The duration of the “last interval” differed considerably between studies and individuals (up to a time interval of 4.5 years before ART initiation; Table S8.1). Lines join the arms of related studies. This suggests that a higher turnover of the rebound-initiating viral reservoir may contribute to a higher contribution from the plasma virus present immediately before the last ART.

**Fig 8:**
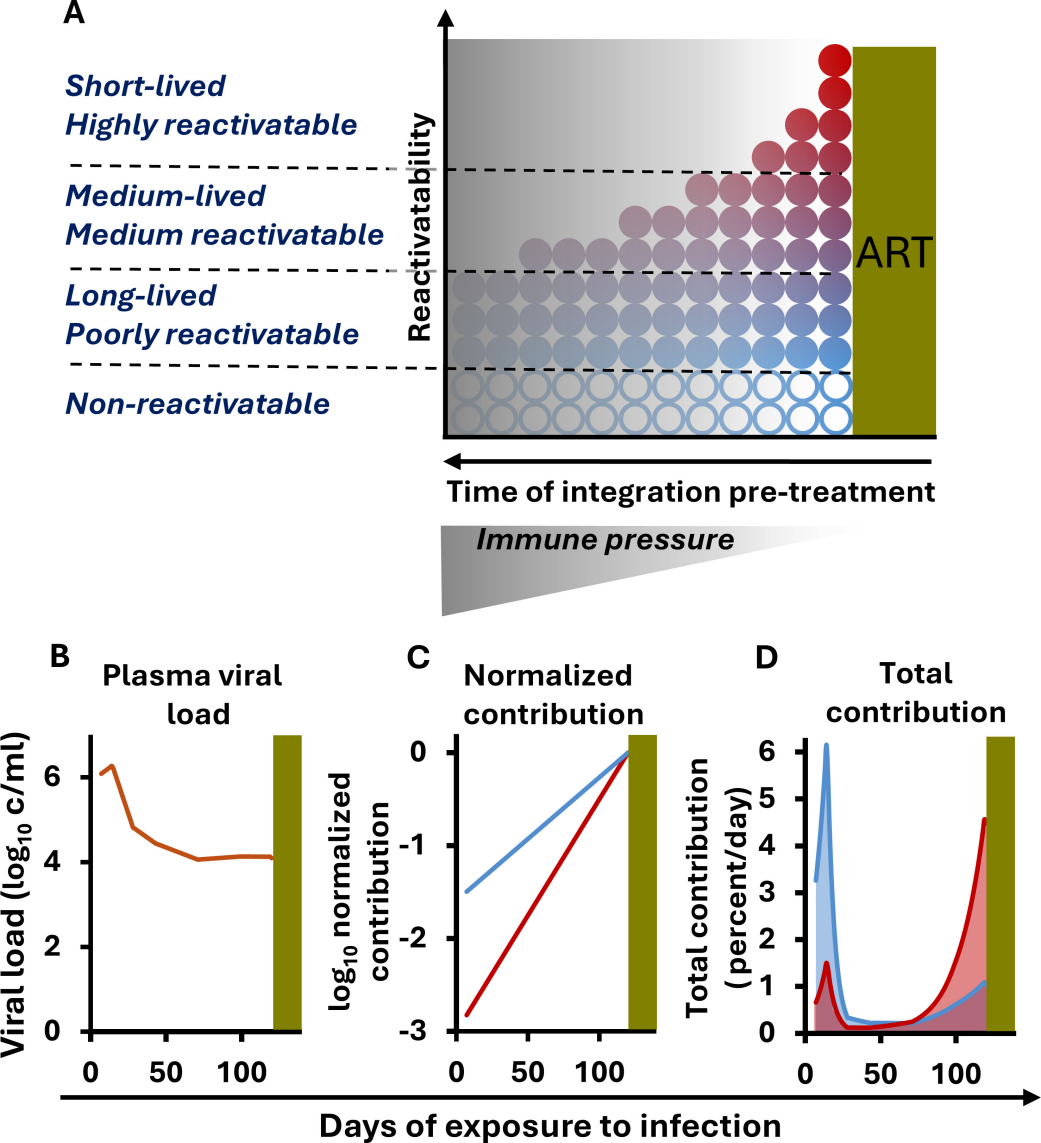
Factors determining reservoir contribution to viral rebound. (**A**) Distribution of reactivation potency at the time of ART. The y-axis illustrates the reactivation potential, that is, the likelihood that a given infected cell will spontaneously contribute to post-treatment viral rebound. When cells are initially infected, a combination of highly reactivatable and non-reactivatable infected cells is laid down. Over time, the most reactivatable cells are lost more quickly. The gray shading on the x-axis illustrates the strength of the immune pressure acting to suppress viral growth. Cells infected just prior to ART initiation have high activation levels and are less affected by the immune pressure due to immune escape mutations. (**B, C, and D**) A schematic illustration of how infected cell activation and immune pressure shape the rebound-initiating infected cell population. (**B**) The mean of log_10_ viral load of RM in Cohort 3. (**C**) The normalized contribution of plasma virus at different times to the RIVR (red) and cell-associated DNA (blue) reservoir. This is normalized to the contribution of plasma virus present at the time of treatment initiation and reflects the estimated decay of the different reservoirs over time (estimated by fitting a log-linear model to the point estimates of normalized contribution in all cohorts simultaneously; see Supplement Text, section 7 for details). (**D**) Total contribution of plasma virus present at different times to rebound (red) and total proviral DNA (blue).

Our studies in SIV/SHIV infection focus more specifically on the source of the rebound plasma virus sampled after treatment interruption, which we term the RIVR. We find that between 65% and 95% of the rebounding virus was associated with plasma virus replicating at ART initiation. By contrast, the viruses replicating at ART initiation contribute a much smaller fraction of the total proviral DNA reservoir. To compare the temporal origins of the reservoir defined in different ways, we combined our data with the available published data on the origins of the reservoir in HIV and SIV/SHIV (Fig. 7). We observed that across these studies the pre-ART timepoint is estimated to contribute around 40%–60% to the total proviral DNA and slightly more to the *in vitro*-reactivated virus (48%–89%). The pre-ART time point makes the largest contribution to the rebounding virus (75%–80%) (Fig. 7). This suggests an apparent gradient in the contribution of the pre-ART time point, dependent on how one measures the different potential reservoirs. This might be explained by the relative susceptibility of these reservoir populations to reactivation, with more “reactivation-prone” provirus (i.e*., ex vivo* reactivable or spontaneously reactivating *in vivo* during ATI) turning over more rapidly during untreated infection (hence, earlier time points are effectively purged from the reservoir). By contrast, total proviral DNA also includes relatively stable provirus that persists for longer (leading to a lower normalized contribution of later time points) (Fig. 8). This suggests a potential hierarchy of proviral activation potential, with spontaneously activatable virus populations (contributing to post-treatment rebound) being more active than *ex vivo*-inducible virus populations, which, in turn, are more active than total proviral DNA. While there are significant caveats in directly comparing the different studies (which differ in a number of factors including timing of plasma virus samples and how relationships between viral populations were estimated), it does suggest that the way we measure and define the reservoir may be associated with the decay rate of that population of cells, and the timing of the seeding of these reservoirs then follows the same pattern.

Another explanation for the recent origins of the rebound-initiating viral reservoir is immune sieving. That is, active infection involves a cat-and-mouse process of immune recognition and viral escape mutation. The most recently replicating virus prior to ART initiation represents the virus capable of replicating under contemporaneous immune pressure, whereas older viruses may be recognized and controlled (although this is unlikely in day 14 treated monkeys). Accordingly, the most recent and more immune-escaped virus had an advantage during the process of rebound (which likely occurs under similar immune pressures to those present at ART initiation). We have recently shown, using data from Cohort 1, that the same CD8+ T cell escape mutations seen at the end of the first treatment interruption are also seen to dominate the second viral rebound (as well as the same viral barcodes) (39). Subsequent CD8+ T cell depletion led to the emergence of wild-type (non-escaped) viral sequences, suggesting that the dominance of escaped clones was driven by CD8+ T cell immune pressure. In unpublished data (38) on Cohort 3, we see that the viruses replicating at ART initiation and those rebounding at ATI are resistant to contemporaneous autologous neutralizing antibodies. By contrast, plasma virus from the peak of infection (day 14) and the majority of reservoir proviruses that resemble peak viremia are highly sensitive to the plasma antibodies present at either ART initiation or at rebound. By studying the virus that successfully rebounds after treatment interruption, we have focused on the virus capable of replicating under immune pressure. By contrast, most of the provirus or *in vitro*-reactivated virus detected will be susceptible to host immune control and unable to rebound successfully. For example, Bertagnolli and colleagues have shown that >80% of *ex vivo* reactivation is inhibited by autologous antibodies (44). It is likely that both the intrinsic activation potential and the potential for immune escape are factors driving the major contribution of plasma virus present around ART initiation to the post-treatment rebound virus (Fig. 8).

Our study provides direct comparisons of the plasma virus rebounding at ART interruption, the plasma virus replicating before ART initiation, and the total proviral DNA reservoir on ART before and after ART interruption in cohorts of monkeys treated at different times post-infection (on days 14, 42, and 120 post-infection). In addition, macaques in two out of three cohorts underwent a second round of antiretroviral treatment and interruption, which allowed us to study the effect of treatment interruption on the latent reservoir. This study differs from similar studies of the HIV reservoir (23–31) in several ways. First, the identity and origins of virus were monitored not by phylogenetic relationships (as used in studies of HIV) but through the identity of barcoded virus, providing both a deep and quantitative measure of individual viral lineages. Our modeling to predict the origins of virus used cross-sectional frequencies of barcodes to estimate the contribution of different time points, whereas other studies have attempted to model the phylogenetic evolution of virus to identify the timing of sequence origin (24, 25, 27, 31, 41). A major advantage of the use of an animal model of barcoded virus infection is the ability to trace and compare viral lineages, as well as to sample virus at regular time points, including soon after viral rebound.

There are also important limitations in extrapolating from our study of SIV/SHIV to understand HIV latency and rebound. First, the natural history of infection with SIV and SHIV differs. Second, infected animals commenced ART very early in infection compared to most research studies of PLWH and clinical practice (although current guidelines recommend ART as soon after diagnosis as possible). Finally, ART duration was also considerably shorter than that in most studies of PWH. Our analysis was a post-hoc analysis of samples from animals from three unrelated studies of SIV/SHIV persistence and included animals treated with different interventions and ART durations. However, analysis of only the controls from each cohort showed very similar results (Table S9.1).

Our analysis suggests that the virus reservoir population that drives post-treatment rebound is largely formed around the time of ART initiation, consistent with previous studies (32, 34). In addition, we show that during sequential treatment interruption and ART initiation, the RIVR appears to be reformed each time, with the majority of rebound virus originating from cells infected at the time of initiation of the most recent ART. While this observation needs validation in clinical studies of HIV treatment and rebound, if confirmed, it suggests that ATIs may have clinical relevance even if they do not substantially shift the proviral DNA or *ex vivo*-reactivatable reservoir by influencing the RIVR (19, 45). In addition, targeting the formation of the reservoir around the time of ART initiation may produce major reductions in the rebound-initiating reservoir and reductions in the frequency of viral rebound (46). Our study also suggests that analysis of the proviral DNA is of limited utility in predicting the virus that will rebound after treatment interruption, likely because the majority of the proviral DNA is either quiescent and unable to initiate viral rebound or is effectively targeted by host immunity. However, this also suggests that rebound is typically initiated by a very small fraction of the cell-associated viral DNA reservoir. Therefore, measuring the impact of “reservoir purging” interventions by measuring changes in the total DNA levels might significantly underestimate their effects on viral rebound (e.g., even if one eliminates the majority of the RIVR, it may have relatively little effect on the total DNA). Methods to characterize and measure the rebound-initiating viral reservoir would greatly accelerate studies of HIV/SIV latency and rebound.

## MATERIALS AND METHODS

### Animal models of SIV and SHIV infections

#### Cohort 1

Fourteen male Mamu-A*01 + rhesus macaques (Macaca mulatta), 46–129 months of age, were infected intravenously with 10,000 infectious units (IU; determined in TZMbl cells) of SIVmac239M, a genetically barcoded virus with a 34-base genetic barcode inserted between the vpx and vpr accessory genes of the infectious molecular clone (36). On day 14 post-infection, all animals were treated subcutaneously with TDF (5.1 mg/kg), FTC (40 mg/kg), and DTG (2.5 mg/kg) as a single daily injection at 1 mL/kg. ART was maintained for 205 or 206 days before macaques underwent ATI that lasted for 61 or 63 days. FTY720 was orally administered to half the animals at a dose of 500 μg/kg per day starting 1 month prior to ART interruption for a total of 4 months (Fig. 1A). All animals were subjected to a second cycle of ART lasting 93 days before the second ATI (39).

#### Barcode sequencing

Extraction of RNA from plasma samples was done using the QIAGEN EZ1 Virus Mini Kit v2.0, and cDNA synthesis was performed with Superscript III reverse transcriptase using the reverse primer SL8R1 (5′-AGC TGA GAG AGG ATT TCC TCCC-3′). Isolation of DNA from mononuclear cells was done using the QIAamp DNA Blood Mini Kit. The number of input templates was quantified by qRT-PCR with SYBR Green using primers VpxF1 (5′-CTA GGG GAA GGA CAT GGG GCA GG-3′) and VprR1 (5′-CCA GAA CCT CCA CTA CCC ATT CATC-3′).

Target regions were amplified by PCR, with MiSeq adaptors added using the forward primer VpxF1.Illumina (5′-TCG TCG GCA GCG TCA GAT GTG TAT AAG AGA CAG CTA GGG GAA GGA CAT GGG GCA GG-3′) and reverse primer SL8R1.Illumina (5′-GTC TCG TGG GCT CGG AGA TGT GTA TAA GAG ACA GAG CTG AGA GAG GAT TTC CTC CC-3′).

Amplified products were pooled and purified by gel extraction using the QIAquick Gel Extraction Kit to remove primer dimers, and sequencing was performed using an Illumina next-generation sequencing platform.

Reads were filtered to exclude those with barcodes not matching the original stock, the absence of the Tat-SL8 epitope, or the presence of insertions or deletions within that region.

Because template numbers and amplification efficiency varied across time points, sequencing runs were excluded if they contained fewer than 1,000 RNA templates (200 for DNA) or fewer than 200 RNA reads (100 for DNA) after filtering.

The limit of detection was determined for each run based on template input and read depth (Text S1), and due to variability in detection sensitivity across barcodes and Tat-SL8 variants over time, background subtraction was applied to all detected barcodes, Tat-SL8 variants, and their combinations unless otherwise specified.

#### Cohort 2

Six rhesus macaques, which were negative for the Mamu-B*08 and -B*17 major histocompatibility complex (MHC) alleles, were assigned to this study at Emory National Primate Research Center under Institutional Animal Care and Use Committee (IACUC) protocols 2003470 and 201700224. RMs were i.v. infected with 10,000 IU of SIVmac239M and initiated daily, subcutaneously injected ART (2.5 mg/kg DTG, 40 mg/kg FTC, and 5.1 mg/kg TDF) at 6 weeks post-infection. Three RMs were administered an investigational experimental immunomodulator studied for latency reversal properties following ART initiation (manuscript in preparation) (Fig. 1B). ATI was performed after 370 days of ART, and RMs were euthanized within 10 weeks following ATI.

#### Barcode sequencing

RNA was extracted from plasma using the QIAamp Viral RNA Mini Kit and eluted in 65 μL buffer. cDNA was generated using Superscript III reverse transcriptase with a Vpr-specific reverse primer (Vpr.cDNA3: 5′-CAG GTT GGC CGA TTC TGG AGT GGA TGC-3′ at positions 6406–6380), followed by stepwise incubation and RNaseH treatment. The resulting cDNA was quantified by qRT-PCR using VpxF1 and VprR1 primers (VpxF1 5′-CTA GGG GAA GGA

CAT GGG GCA GG-3′ at 6082–6101 and VprR1 5′-CCA GAA CCT CCA CTA CCC ATT CATC-3′ at 6220–6199). Target sequences were subsequently amplified by PCR with high-fidelity polymerase, simultaneously incorporating MiSeq adapters and unique 8-nucleotide index sequences for multiplexed sequencing. Sequencing was performed on Illumina’s MiSeq instruments.

#### Cohort 3

Eighteen rhesus macaques were infected with 1 × 10^6^ IU of barcoded SHIV.D. Macaques were treated with the combinational ART (DTG, FTC, and TDF) at 120 or 121 days post-infection. The ART was maintained for 178 or 194 days before the first ATI. At the first ATI, nine RMs received a single 30 mg/kg i.v. dose of VRC07.523.LS, while the other nine RMs remained untreated. ART was re-initiated 120–121 days after the first ATI initiation and maintained for 161–189 days before the second ATI. Immediately before the second ATI, all animals were treated with both 30 mg/kg VRC07.523.LS and VRC01.LS (Fig. 1C). All macaques were subjected to elective necropsy 5 months after the last ATI.

#### Barcode sequencing

RNA was extracted from plasma using the QIAamp Viral RNA Mini Kit and eluted in 65 μL buffer. cDNA was generated using Superscript III reverse transcriptase with a Vpr-specific reverse primer (Vpr.cDNA3: 5′-CAG GTT GGC CGA TTC TGG AGT GGA TGC-3′ at positions 6406–6380), followed by stepwise incubation and RNaseH treatment. The resulting cDNA was quantified by qRT-PCR using VpxF1 and VprR1 primers (VpxF1 5′-CTA GGG GAA GGA

CAT GGG GCA GG-3′ at 6082–6101 and VprR1 5′-CCA GAA CCT CCA CTA CCC ATT CATC-3′ at 6220–6199). Target sequences were subsequently amplified by PCR with high-fidelity polymerase, simultaneously incorporating MiSeq adapters and unique 8-nucleotide index sequences for multiplexed sequencing. Sequencing was performed on Illumina’s MiSeq instruments.

For low-template samples input, single-genome amplification followed by direct Sanger sequencing was employed to determine the frequency and diversity of unique barcodes. cDNA synthesis and PCR were conducted as described above, with the exception that cDNA was first diluted to limiting concentrations prior to amplification.

Sequences derived from infected animals were compared to a reference set of 14,357 barcodes from the original viral stock, with only exact matches considered valid input sequences. Due to the short infection period prior to cART initiation and the small insert size, the majority of recovered sequences corresponded directly to known barcodes. A small proportion of barcodes contained deletions within the insert, resulting in partial inclusion of the adjacent vpx sequence; although these represented only ~0.7% of all inserts, they were retained as distinct, genetically identifiable variants. Based on the minimum number of sequences that would result from a single copy of an input template, a sample-specific theoretical limit of detection was established, and sequences falling below this threshold were excluded from analysis (36, 38).

### Mathematical analysis

#### Modeling the contribution of plasma virus to viral rebound and proviral DNA

We developed a mathematical model that uses the viral load of the barcode in plasma before ART initiation to estimate two outcomes: the probability of reactivation shortly after treatment interruption or the probability of observing this barcode in total proviral DNA samples.

We analyze whether the viral loads of barcodes in plasma at a particular time are predictive of which barcodes are seen in plasma during ATI. We model reactivation events using a Poisson distribution, which defines the probability of a given number of independent events occurring at a constant rate within a time window.

Let *r_k_* denote the reactivation rate per day per viral copy for subject *k*, and *w_k_* denote the time window during which reactivation of barcodes may be detected *k* = 1,…,*N_s_*. Let *v_k_*_*ij*_ represent the viral load of *i*-th barcode at the *j*-th measurement for subject *k,* where *j* = 1,…,*N_m_*. Using these notations, we derive the probability (*P*) of observing at least one reactivation (or the detection of total proviral DNA) of the barcode *i* in subject *k* within the window *w_k_*:


(1) $$P\left(v_{ki1},\ldots ,v_{kiN_{m}}\right)=1-e^{-r_{k}w_{k}\sum_{j=1}^{N_{m}}\alpha_{j}v_{kj}}$$

In order to allow variation in the relative contribution of plasma virus at different time points to the rebound-initiating reservoir or proviral DNA, we introduced weighting parameters $\alpha_{j}$, where $\sum_{j=1}^{N_{m}}\alpha_{j}=1$ is the relative contribution of the plasma virus at measure *j* to the reactivatable pool. We assume that all weights $\alpha_{j}$ are equal across subjects for measurement at the same time.

Given that the outcome variable $y_{i}$ is binary (i.e., can take two values: 0, the barcode did not rebound and 1, the barcode rebounded), we model it as a Bernoulli random variable. Substituting the probability from equation 1 into the Bernoulli probability mass function and then taking the natural logarithm of this expression yields the log-likelihood function equation 2:


(2) $$\begin{array}{rl} & LL\left(\beta_{1},\ldots ,\beta_{N_{s}},\alpha_{1},\ldots ,\alpha_{N_{m}}\right)=\\ & \;=\sum_{k=1}^{N_{s}}\sum_{i=1}^{N_{b}}(y_{ki}\ln P\left(v_{ki1},\ldots ,v_{kiN_{m}}\right)+\left(1-y_{ki}\right)\ln\left(1-P\left(v_{ki1},\ldots ,v_{kiN_{m}}\right)\right) \end{array}$$

where *y_i_* = 1 if barcode *i* rebounded or was present in the total cell-associated DNA and 0 if neither occurred, *N_b_* is the total number of barcodes, and $\beta_{k}=r_{k}\;w_{k}$. For the barcode viral loads that were below the detection threshold, we assigned the detection threshold value.

For the convenience of the presentation, we transformed the relative contributions $\alpha_{i}$ to normalized contributions $\gamma_{j}=\frac{\alpha_{j}}{\alpha_{N_{m}}}$. The total contribution (in percent) to the rebound or the total latent reservoir from the *i*-th measurement is defined as the following $\delta_{kj}=\frac{100\;\alpha_{j}V_{kj}}{\sum_{j=1}^{N_{m}}\alpha_{j}V_{kj}},$ where *V_kj_* is the viral load of the *k*-th subject at the *j*-th measurement.

The fitting procedure was implemented in Wolfram Mathematica 12.3.

## Data Availability

The data and analysis used in the current study are available on the GitHub repository via the link https://github.com/iap-sydney/Temporal_origin_of_the_SIV_SHIV_rebound.git.

## SUPPLEMENTAL MATERIAL

Cohort 1 - barcodes jvi.00366-26-s0001.xlsx Viral loads for each barcode in plasma samples and the fraction of each barcode in PBMC and lymph node samples for Cohort 1.

Cohort 2 - barcodes jvi.00366-26-s0002.xlsx Viral loads for each barcode in plasma samples for Cohort 2.

Cohort 3 - barcodes jvi.00366-26-s0003.xlsx Viral loads for each barcode in plasma samples and the fraction of each barcode in PBMC samples for Cohort 3.

Supplemental material jvi.00366-26-s0004.pdf Detailed summary of macaque cohorts, as well as figures and tables with additional data analysis.

ASM does not own the copyrights to Supplemental Material that may be linked to, or accessed through, an article. The authors have granted ASM a non-exclusive, world-wide license to publish the Supplemental Material files. Please contact the corresponding author directly for reuse.
