# Supplementary material for: The rebound-initiating viral reservoir (RIVR) in SIV/SHIV infection is dominated by plasma virus sequences present at the time of ART initiation
Source: J Virol. 2026 Jul 28;100(8):e00366-26. doi: 10.1128/jvi.00366-26 (PMC13483457; doi:10.1128/jvi.00366-26)
Supplement: Supplementary file 1 [file jvi.00366-26-s0004.pdf]

Supplementary section 1. Summary of cohorts.

Virus	Virus dose	Treatment			N	Monkey's IDs	Comments	Reference/Ethics policy
		Initiation (days post infection)	Regimen	Duration (days)				
**Emory National Primate Research Center (Cohort 1)**								
SIVmac239M (Barcoded)	1x10^4 IU	1st ART – day 14,<br><br>2nd ART – day 281 or 282	FTC, TDF, DTG	1st ART – 205 or 206 days,<br><br>2nd ART – 93 days	14	RMf16, RRf12, RUh14, RYm15, 13D064, 14_10, RFb16, RLm14, RNd15, RYk16, 14C207, 9_047, REe16, RJy13	RLm14, RNd15, RYk16, 14C207, 9_047, REe16, RJy13 are controls and RMf16, RRf12, RUh14, RYm15, 13D064, 14_10, RFb16, are treated with Fingolimod (FTY720) on day 189 or 190 to days 309 or 310 post infection.	Emory IACUC PROTO201700655, (1)
**Emory National Primate Research Center (Cohort 2)**								
SIVmac239M (Barcoded)	1x10^4 IU	Day 42	FTC, DTF, DTG	370 days	6	13C339, 13D075, RLy16, REn16, RTn16, 14C333,	Control animals (13C339, 13D075, RLy16) and animals treated with an experimental immunomodulator at ATR-initiation (REn16, RTn16, 14C333).	IACUC 2003470, 201700224
**Emory National Primate Research Center (Cohort 3)**								
TF-SHIV.D.191859 (Barcoded)	1x10^6 IU	1st ART – day 120 or 121	FTC, DTF, DTG	1st ART – 178 or 194 days	18	RZn17, RSk17, RIa17, RVg17, RFb17, RGj17, REf17, 34534, RHe17, RTb17, 14D076, RSd17, RNc17, RZe17, RYi17, RFa17, RRj17, RBm17	Animals RTb17, 14D076, RSd17, RNc17, RZe17, RYi17, RFa17, RRj17, RBm17 were injected with 30mg/kg of VRC07-523LS on the	(2)

		2nd ART – day 419 or 435		2nd ART - 161 or 189 days			first day of the 1st ART interruption (day 298 or day 315). All animals were injected with 30mg/kg of both VRC07-523LS and VRC01-LS on the 1st day of the 2nd ART interruption (days 596, 601, 606, 608)	

Supplementary Table S1.1. Summary of the cohorts.

Supplementary section 2. Supplement to Fig. 3.

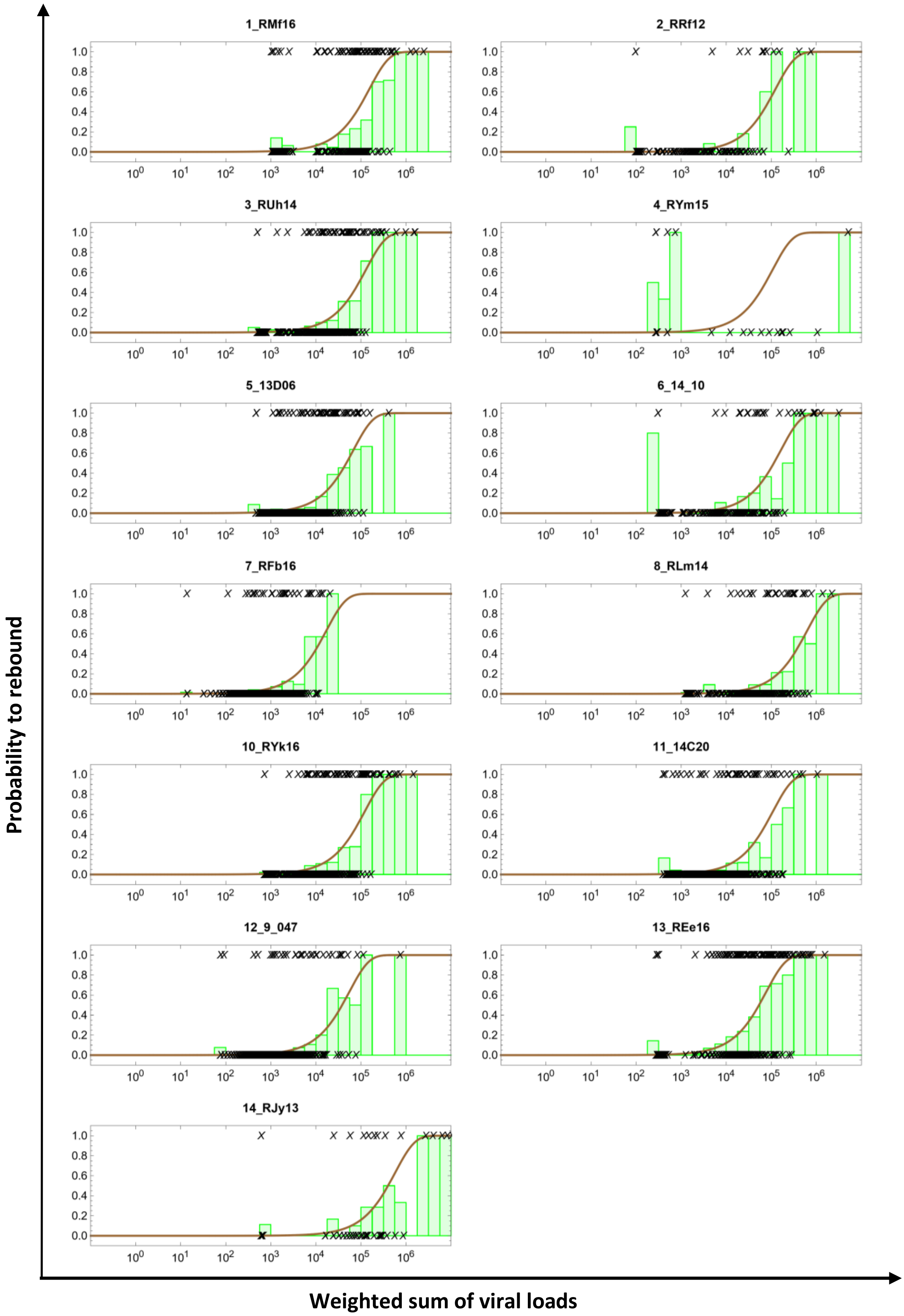

Supplementary Fig. S2.1. **The best-fit of the model (Equation (2) in Materials and Methods) estimating the contribution from plasma virus at different timepoints <u>pre-ART to the probability of rebound at the first ATI in Cohort 1</u> (supplement to Fig. 3A).** The x-axis is the sum of viral loads from predictor measurements

weighted by the per-virion contribution of respective measurements. The horizontal position of black "x" symbols indicates the weighted sum of viral load for given barcodes. Barcodes that did not rebound have y-coordinate = 0, and barcodes that rebounded have y-coordinate = 1. The green-shaded bars are the fractions of barcodes that rebounded in the given viral load bin. The best-fit parameters and the percent total contribution to the reactivatable pool are in Supplementary Table S2.1.

	α_1 (Day 7)	α_2(Day 14)
**Cohort 1**	0.658698	0.341302

ID	β	Percent total contribution	
		**Day 7**	**Day 14**
1_RMf16	6.43E-06	1.56768	98.4323
2_RRf12	7.98E-06	0.446699	99.5533
3_RUh14	7.65E-06	1.28346	98.7165
4_Rym15	9.22E-06	0.0349099	99.9651
5_13D06	1.46326E-05	14.4072	85.5928
6_14_10	6.08E-06	1.65227	98.3477
7_RFb16	5.85058E-05	59.8457	40.1543
8_RLm14	1.59E-06	1.44098	98.559
10_Ryk16	8.18E-06	2.1712	97.8288
11_14C20	9.14E-06	5.60481	94.3952
12_9_047	1.81115E-05	20.2442	79.7558
13_Ree16	1.29528E-05	0.738144	99.2619
14_Rjy13	1.73E-06	0.041628	99.9584

Supplementary Table S2.1. The best-fit parameters of the model presented in Equation (2) of Materials and Methods, estimating the percent of the total contribution from plasma virus at different timepoints <u>pre-ART to predicting rebound at the first ATI in Cohort 1</u> (supplement to Fig. 3A and Supplementary Fig. S2.1).

Supplementary section 2. Supplement to Fig. 3.

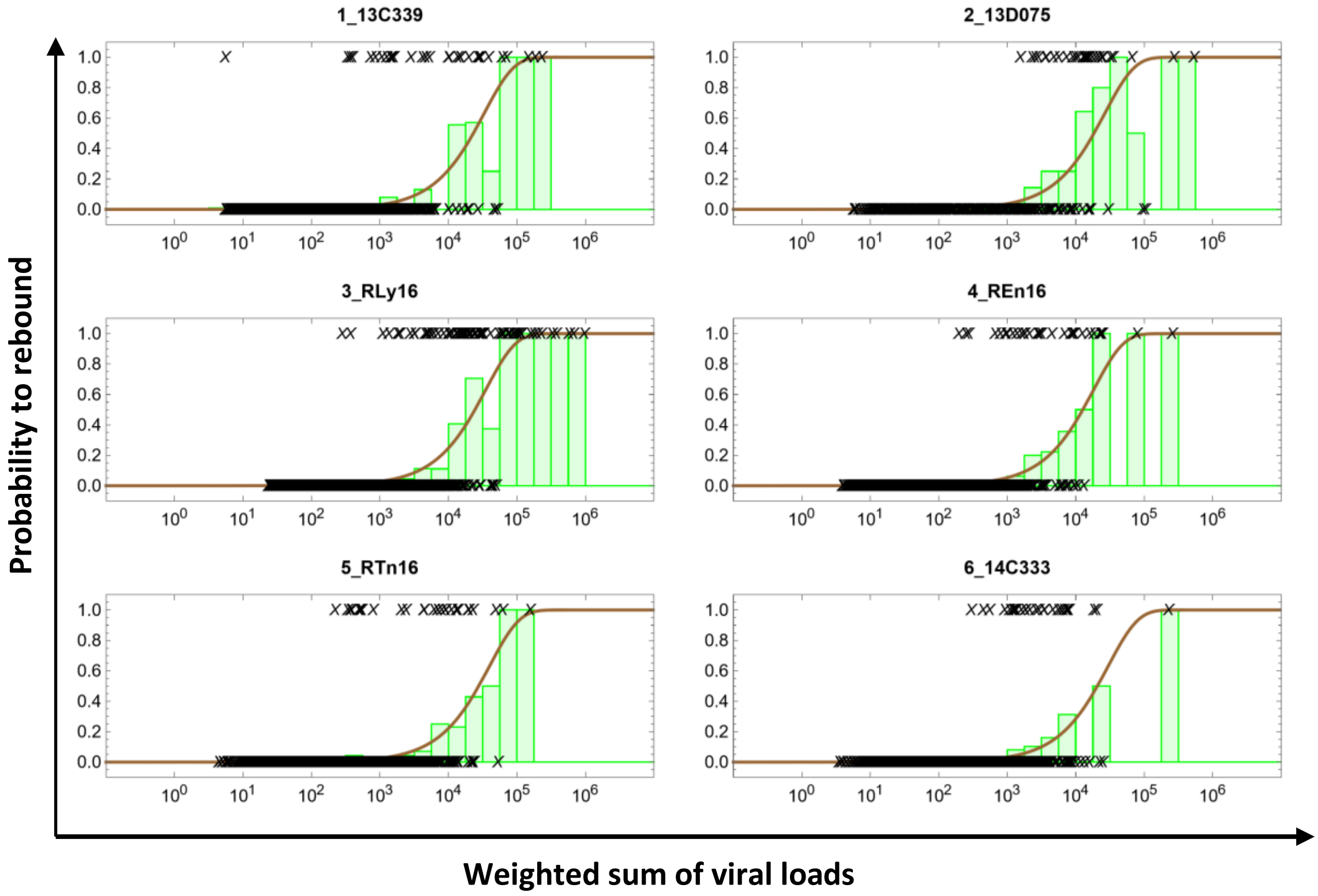

Supplementary Fig. 2.2. The best-fit of the model (Equation (2) in Materials and Methods) estimating contribution from plasma virus at different timepoints pre-ART to the probability of rebound at the first ATI in Cohort 2. (supplement to Fig. 3B). The x-axis is the sum of viral loads from predictor measurements weighted by per-virion contribution of respective measurements. The horizontal position of black "x" symbols indicates the weighted sum pre-treatment viral load of different barcodes. Barcodes that did not rebound have y-coordinate = 0, and barcodes that rebounded have y-coordinate =1. The green-shaded bar is the fraction of barcodes (in the given bin) that rebounded. The best-fit parameters and the percent total contribution to the reactivatable pool are in Supplementary Table S2.2.

Supplementary section 2. Supplement to Fig. 3.

	α_1(Day 7)	α_2(Day 14)	α_3(Day 42)
**Cohort 2**	0	0.027944	0.972056

		**Percent total contribution**		
**ID**	**β**	**Day 7**	**Day 14**	**Day 42**
1_13C339	3.00009E-05	0.	24.908	75.092
2_13D075	0.000037595	0.	46.7391	53.2609
3_RLy16	2.84207E-05	0.	14.1093	85.8907
4_REn16	5.47046E-05	0.	40.9647	59.0353
5_RTn16	2.50405E-05	0.	33.0914	66.9086
6_14C333	3.34896E-05	0.	52.5091	47.4909

Supplementary Table S2.2. The best-fit parameters of the model presented in Equation (2) of Materials and Methods, estimating the percent of the total contribution from plasma virus at different timepoints pre-ART to predicting rebound at the first ATI in Cohort 2 (supplement to Fig. 3B and Supplementary Fig. S2.2).

Supplementary section 2. Supplement to Fig. 3.

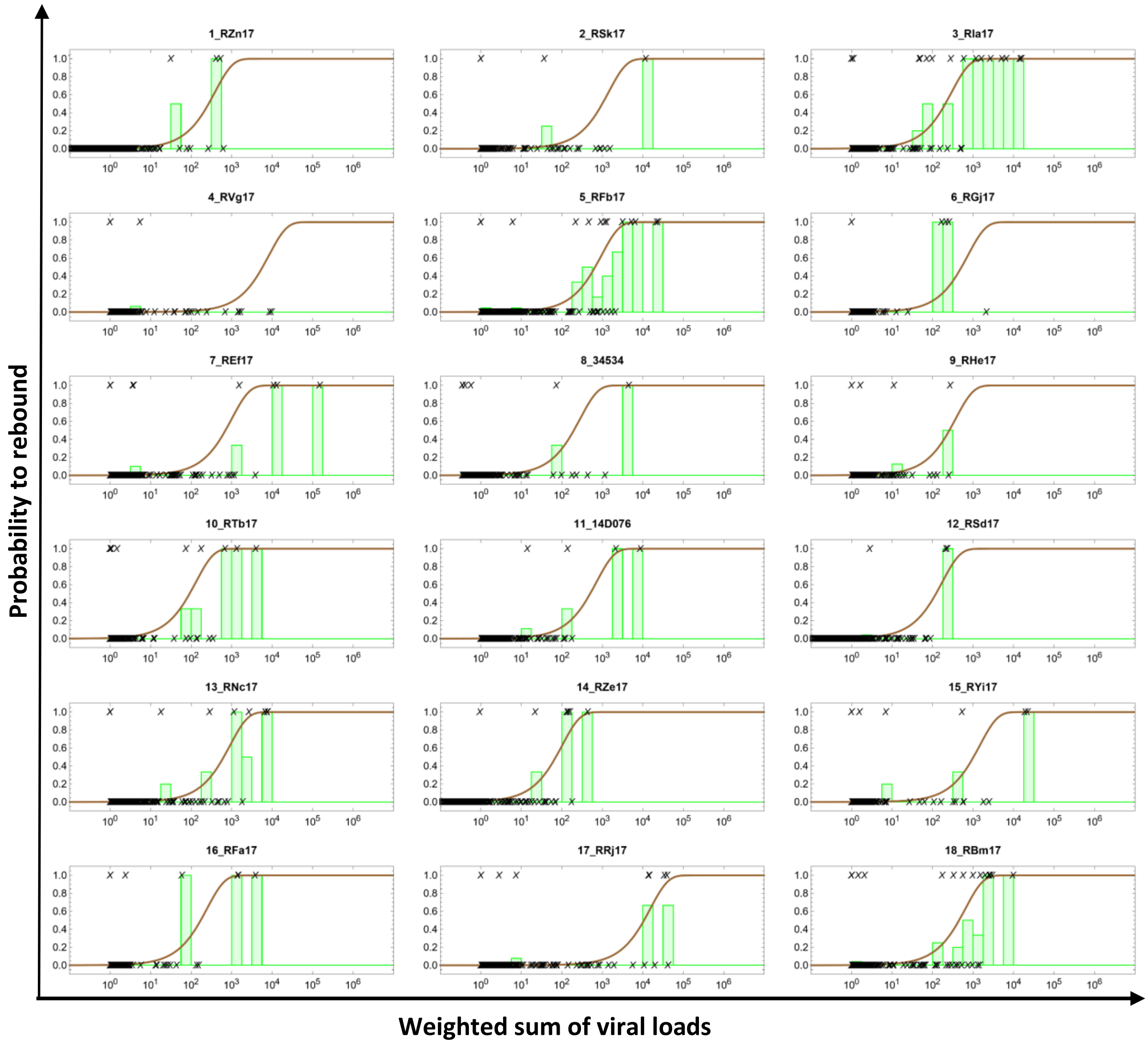

Supplementary Fig. S2.3. The best-fit of the model (Equation (2) in Materials and Methods) estimating contribution from plasma virus at different timepoints pre-ART to the probability of rebound at the first ATI in Cohort 3 (supplement to Fig. 3C). The x-axis is the sum of viral loads from predictor measurements weighted by per-virion contribution of respective measurements. The horizontal position of black "x" symbols indicates the weighted pre-treatment viral load of different barcodes. Barcodes that did not rebound have y-coordinate = 0, and barcodes that rebounded have y-coordinate = 1. The green-shaded bar is the fraction of barcodes (in the given bin) that rebounded. The best-fit parameters and the percent total contribution to the reactivatable pool are in Supplementary Table S2.3.

Supplementary section 2. Supplement to Fig. 3.

	**α_1 (Days 14,15)**	**α_2 (Days 119-121)**
**Cohort 3**	0.000144291	0.999856

**ID**	**β**	**Percent total contribution**	
		**Days 14,15**	**Days 119-121**
1_RZn17	0.0025572	13.9441	86.0559
2_RSk17	0.000694279	0.73553	99.2645
3_Rla17	0.00335442	0.515041	99.485
4_RVg17	0.000119036	1.2739	98.7261
5_RFb17	0.00104363	0.834518	99.1655
6_RGj17	0.00133285	6.75094	93.2491
7_REf17	0.000982214	0.180371	99.8196
8_34534	0.00350735	2.71151	97.2885
9_RHe17	0.00267409	21.6456	78.3544
10_RTb17	0.00755155	1.42657	98.5734
11_14D076	0.0013848	1.81979	98.1802
12_RSd17	0.00571808	26.8128	73.1872
13_RNc17	0.00108318	1.43434	98.5657
14_RZe17	0.00973878	9.40784	90.5922
15_RYi17	0.0006755	0.436516	99.5635
16_RFa17	0.00401969	1.8227	98.1773
17_RRj17	0.0000647549	0.27252	99.7275
18_RBm17	0.00153645	0.896802	99.1032

Supplementary Table S2.3. The best-fit parameters of the model presented in Equation (2) of the Materials and Methods, estimating the percent of the total contribution from plasma virus at different timepoints pre-ART to predicting rebound at the first ATI in Cohort 3 (supplement to Fig. 3C and Supplementary Fig. S2.3).

Supplementary section 3. Supplement to Fig. 4.

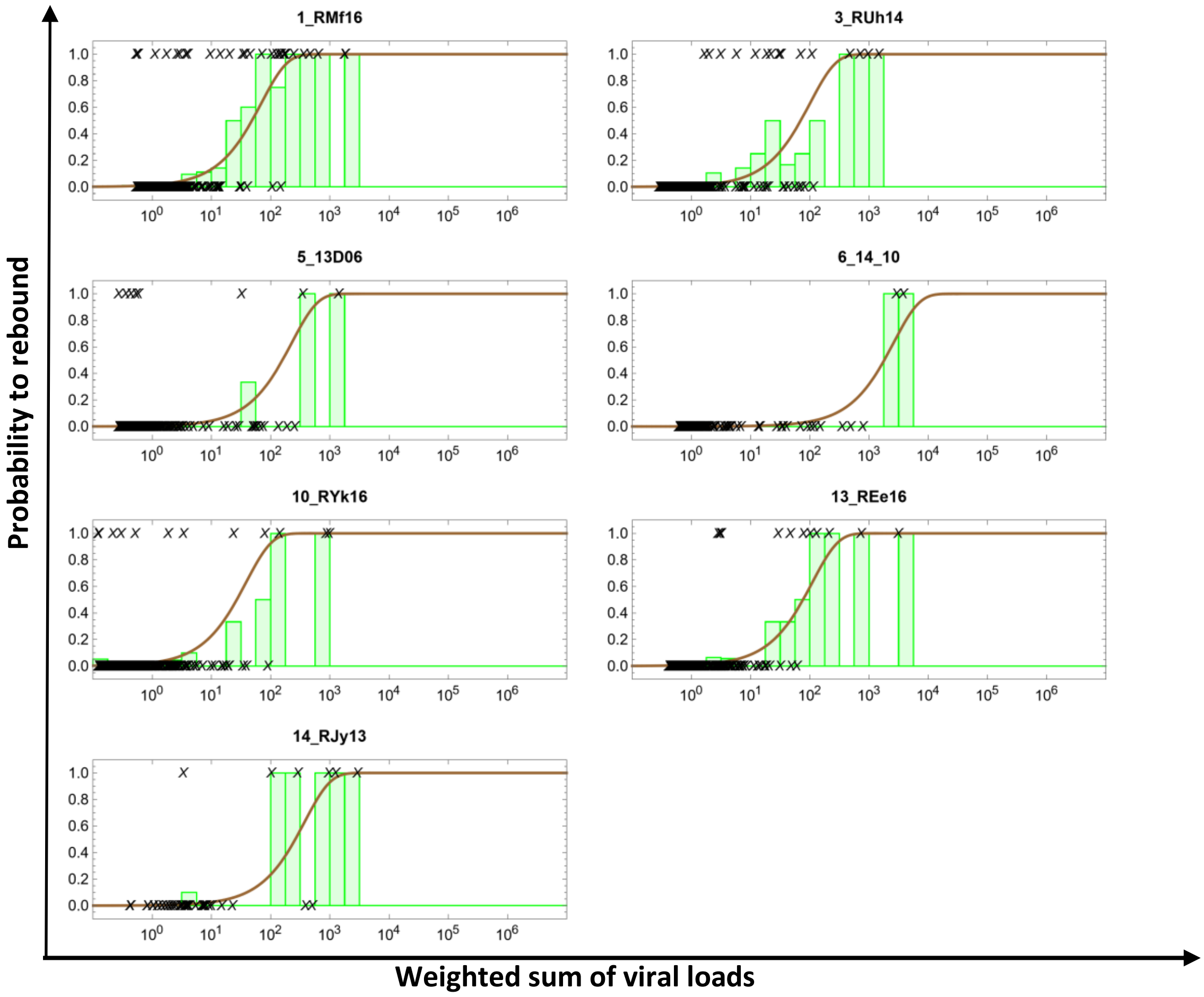

Supplementary Fig. S3.1. The best-fit of the model (Equation (2) in Materials and Methods), estimating contribution from plasma virus at different timepoints at pre-ART and the first ATI to predicting the probability of rebound at the second ATI in Cohort 1 (supplement to Fig. 4A). The x-axis is the total sum of viral loads from predictor measurements weighted by per-virion contribution of respective measurements. The horizontal position of black "x" symbols is the total pre-treatment viral load of different barcodes. Barcodes that did not rebound have y-coordinate = 0, and barcodes that rebounded have y-coordinate =1. The green-shaded bar is the fraction of barcodes (in the given bin) that rebounded. The best-fit parameters and the percent total contribution to the reactivatable pool are in Supplementary Table S3.1.

Supplementary section 3. Supplement to Fig. 4.

		α_1 (Day 7)	α_2 (Day 14)	α_3 (Days 233-239)	α_4 (Days 281-282)
**Cohort 1**		0.0000627098	0.00000875298	0.	0.999929
**ID**	**β**	**Percent total contribution**			
		**Day 7**	**Day 14**	**Days 233-239**	**Days 281-282**
1_RMf16	0.0142774	0.504057	8.52544	0.	90.9705
3_RUh14	0.0098565	0.335968	6.96084	0.	92.7032
5_13D06	0.00436631	2.37805	3.8057	0.	93.8162
6_14_10	0.000383266	0.214999	3.44728	0.	96.3377
10_RYk16	0.0258047	1.05003	12.7445	0.	86.2055
13_REe16	0.00900506	0.260264	9.42784	0.	90.3119
14_RJy13	0.00265221	0.0232015	15.0074	0.	84.9694

Supplementary Table S3.1. The best-fit parameters of the model presented in Equation (2) of Materials and Methods, estimating the percent of the total contribution from plasma virus at different timepoints pre-ART and the first ATI to predicting rebound at the second ATI in Cohort 1 (supplement to Fig. 4A and Supplementary Fig. S3.1).

Supplementary section 3. Supplement to Fig. 4.

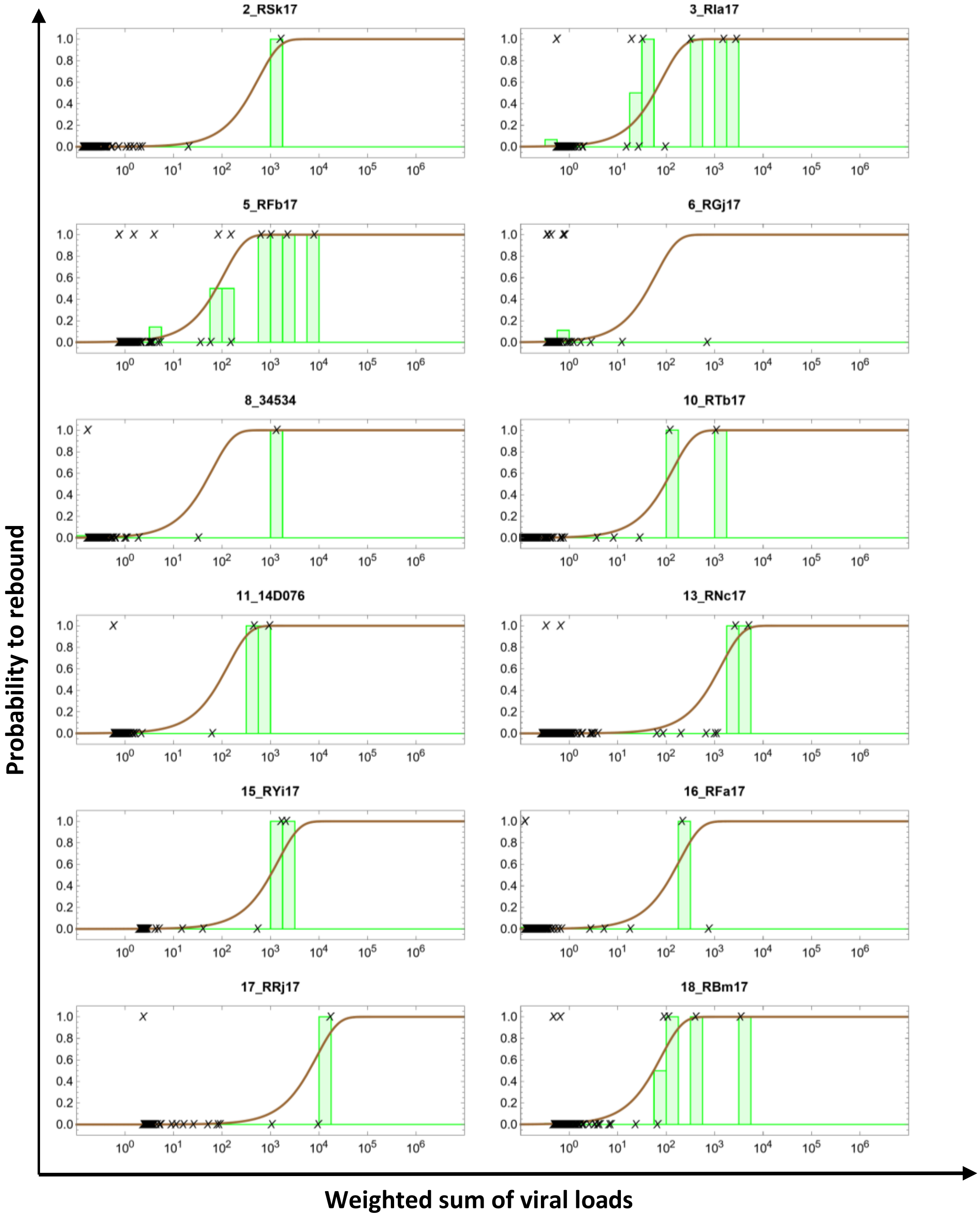

Supplementary Fig. S3.2. <u>The best-fit of the model</u> (Equation (2) in Materials and Methods)estimating contribution from plasma virus at different timepoints <u>pre-ART and the first ATI to predicting the probability of rebound at the second ATI in Cohort 3</u> (supplement to Fig. 4B The x-axis is the sum of viral loads from predictor measurements weighted by per-virion contribution of respective measurements. Horizontal position of black "x" symbols indicates the weighted pre-treatment viral load of different barcodes. Barcodes that did not rebound have y-coordinate = 0, and barcodes that rebounded have y-coordinate =1. The green-shaded bar is the fraction of barcodes (in the given bin) that rebounded. The best-fit parameters and the percent total contribution to the reactivatable pool are in Supplementary Table S3.2.

Supplementary section 3. Supplement to Fig. 4.

		α_1 (Days 14, 15)	α_2 (Days 119-121)	α_3 (Days 313-343)	α_4 (Days 399-416)
**Cohort 3**		0.0000171138	0.00116796	0.0487669	0.950048
**ID**	**β**	**Percent total contribution**			
		**Day 14, 15**	**Day 119-121**	**Days 313-343**	**Days 399-416**
2_RSk17	0.00177137	0.970732	1.29026	2.97714	94.7619
3_RIa17	0.0120427	0.629721	1.19797	3.64901	94.5233
5_RFb17	0.00909588	0.640651	0.749772	3.60425	95.0053
6_RGj17	0.0160632	3.19115	0.434121	2.22352	94.1512
8_34534	0.0158871	1.5857	0.560344	1.27508	96.5789
10_RTb17	0.00733322	1.02581	0.698096	3.99946	94.2766
11_14D076	0.00743533	1.76843	0.939668	8.69501	88.5969
13_RNc17	0.000726027	0.616859	0.417488	0.252187	98.7135
15_RYi17	0.000692099	0.568996	1.27818	4.20478	93.948
16_RFa17	0.00530287	1.86887	0.99142	4.17546	92.9643
17_RRj17	0.00011094	0.216474	0.780199	36.9925	62.0108
18_RBm17	0.0125221	0.799963	0.870651	8.70196	89.6274

Supplementary Table S3.2. The best-fit parameters of the model presented in Equation (2) of Materials and Methods, (2) estimating the percent of the total contribution from plasma virus at different timepoints at pre-ART and the first ATI to predicting rebound at the second ATI in Cohort 3 (supplement to Fig. 3B and Supplementary Fig. S3.2).

Supplementary section 4. Prediction of the rebound at 2nd ATI using plasma virus from the first ATI.

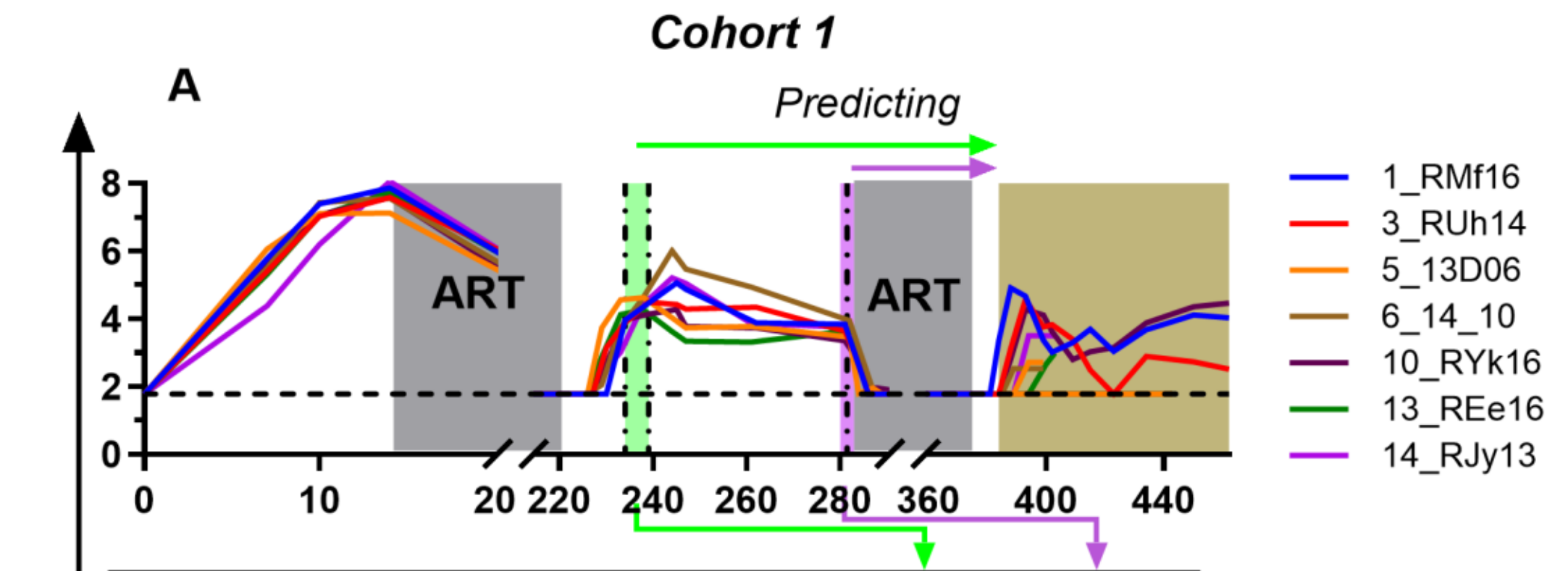

Days of sampling	233-239	281, 282
Mean total contribution (%)	0.26	99.74
Normalised (to days 281, 282) contribution	4.72E-04	1.00

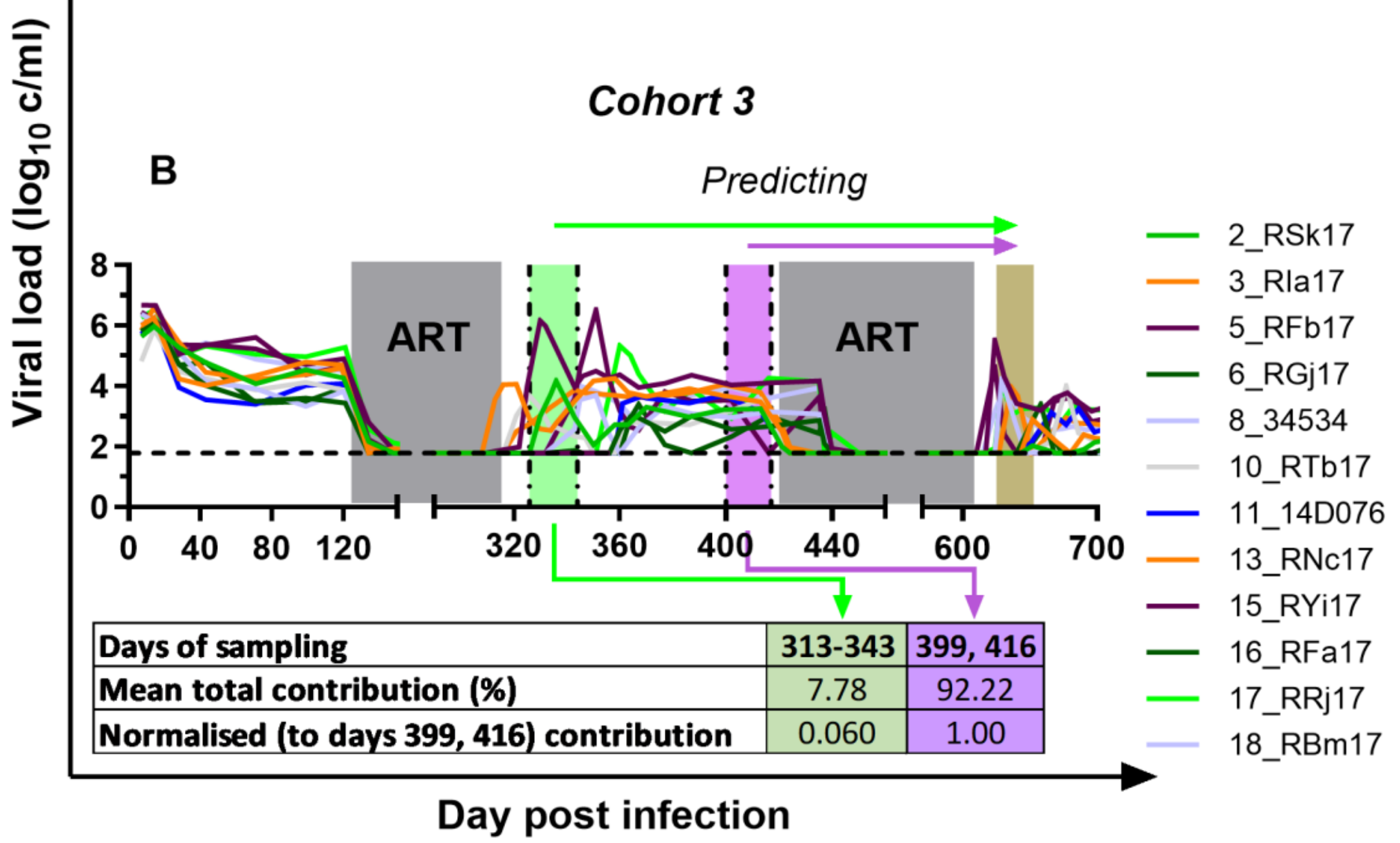

Days of sampling	313-343	399, 416
Mean total contribution (%)	7.78	92.22
Normalised (to days 399, 416) contribution	0.060	1.00

Supplementary Fig. S4.1. Modelling the contribution of the virus at different time points during the first ATI to reactivation at the second ATI. The trajectory of viral load and parameter tables for the macaques of Cohort 1 (A) and Cohort 3 (B). The shaded area between the dash-dotted lines is the interval where sequencing was conducted (different times for different subjects). Khaki-shaded areas are sequencing times after rebound (different for different macaques). The tables below graphs contain contributions to the reactivatable pool derived from the best-fit parameter for the corresponding timepoint (matching colour).

Supplementary section 4. Prediction of the rebound at 2nd ATI using plasma virus from the first ATI.

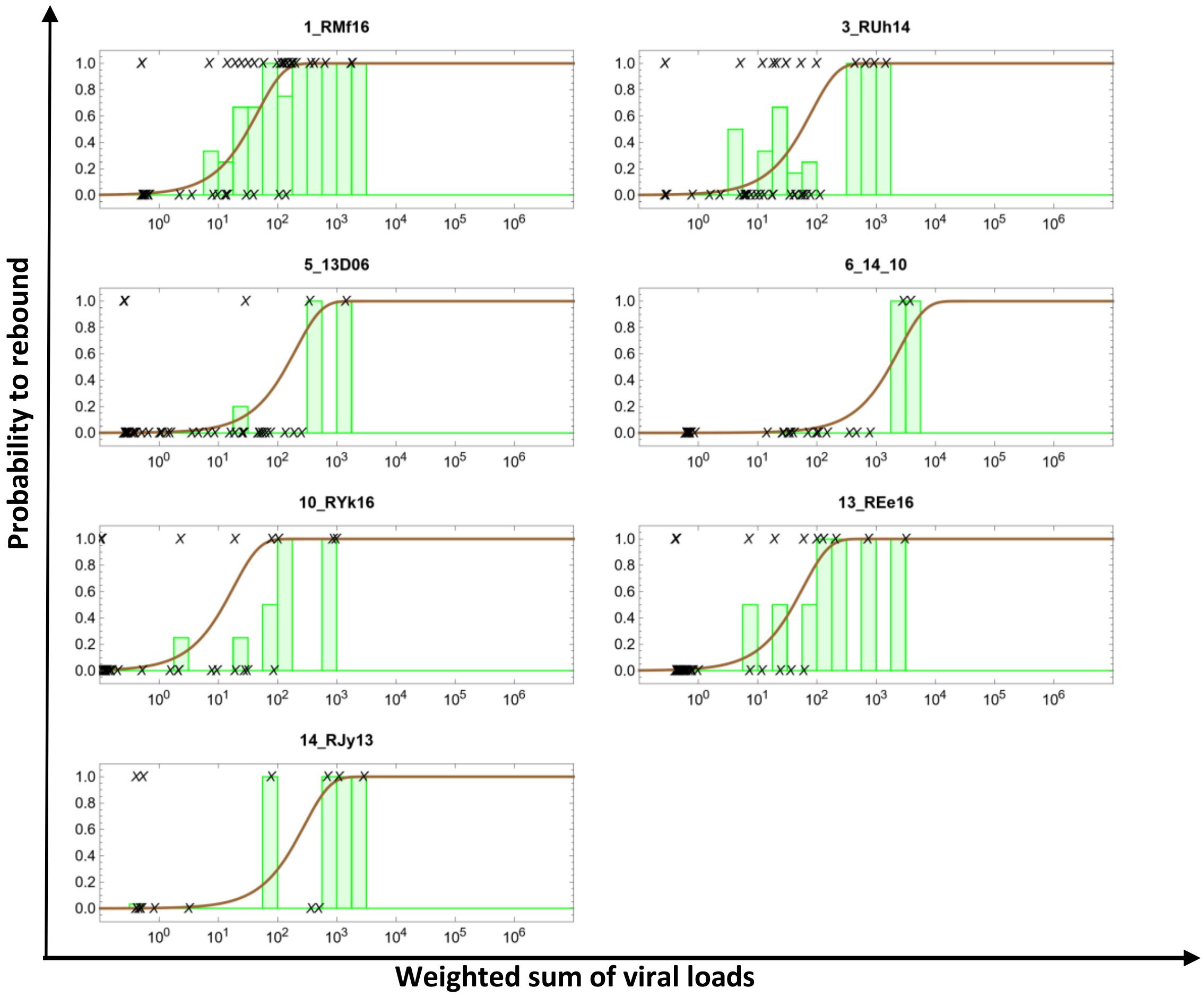

Supplementary Fig. S4.2. The best-fit of the model (Equation (2) in Materials and Methods) estimating contribution from plasma virus at different <u>first ATI timepoints to predicting the probability of rebound at the second ATI in Cohort 1</u> (supplement to Supplementary Fig. S4.1A). The x-axis is the sum of viral loads from predictor measurements weighted by the per-virion contribution of respective measurements. The horizontal position of black "x" symbols indicates the weighted pre-treatment viral load of different barcodes. Barcodes that did not rebound have y-coordinate = 0, and barcodes that rebounded have y-coordinate =1. The green-shaded bar is the fraction of barcodes (in the given bin) that rebounded. The best-fit parameters and the percent total contribution to the reactivatable pool are in Supplementary Table S4.1.

Supplementary section 4. Prediction of the rebound at 2nd ATI using plasma virus from the first ATI.

		α_3 (Days 233-239)	α_4 (Days 281-282)
**Cohort 1**		0.000471643	0.999528
**ID**	**β**	**Percent total contribution**	
		**Days 233-239**	**Days 281-282**
1_RMf16	0.021415	0.199512	99.8005
3_RUh14	0.0122584	0.0826945	99.9173
5_13D06	0.00491054	0.59003	99.41
6_14_10	0.000406658	0.231146	99.7689
10_RYk16	0.0552875	0.288576	99.7114
13_REe16	0.0170072	0.187568	99.8124
14_RJy13	0.00350074	0.229974	99.77

Supplementary Table S4.1. The best-fit parameters of model (2) estimating the percent of the total contribution from plasma virus at different first ATI timepoints to predicting rebound at the second ATI in Cohort 1 (supplement to Supplementary Fig. S4.1A and Supplementary Fig. S4.2).

Supplementary section 4. Prediction of the rebound at 2nd ATI using plasma virus from the first ATI.

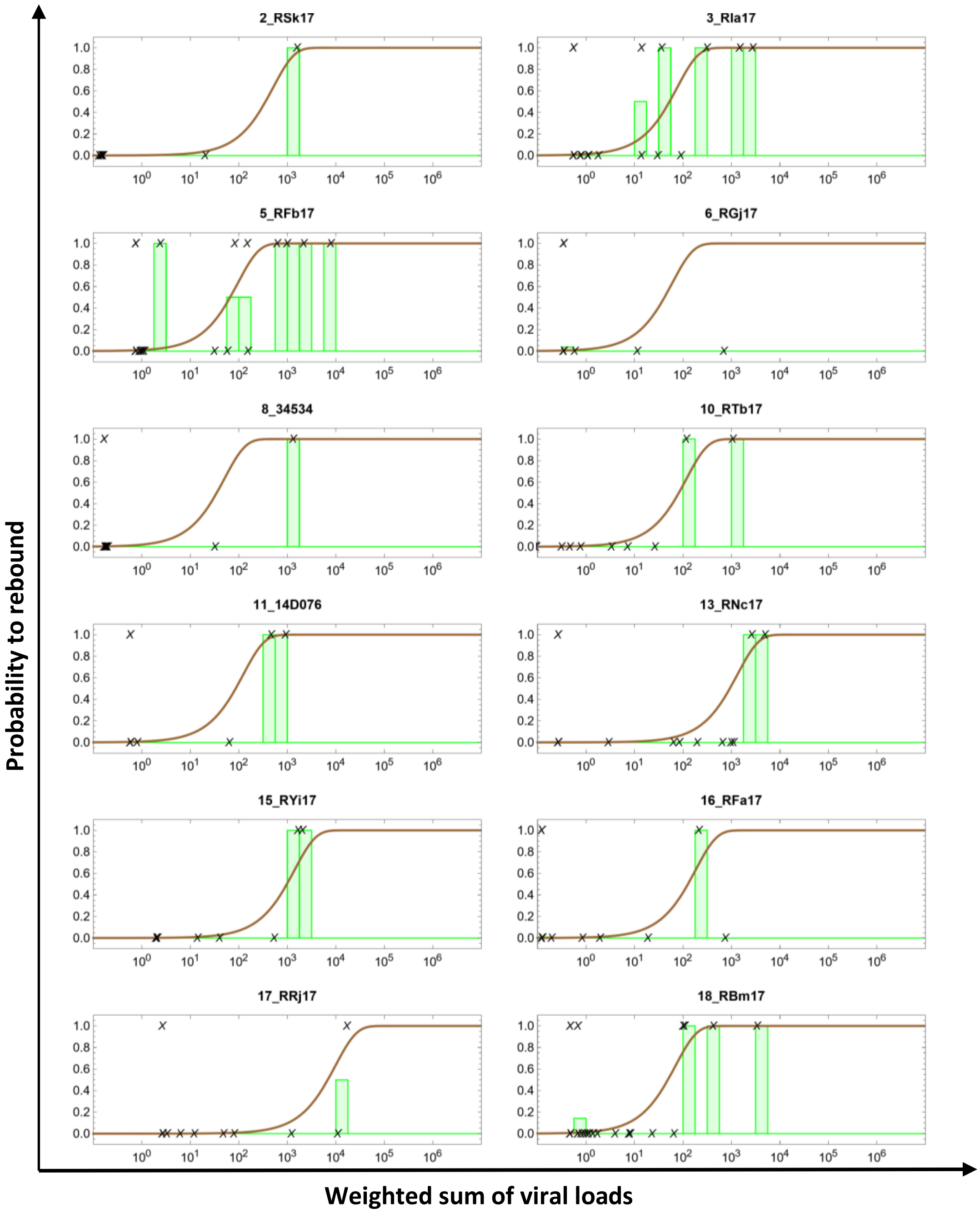

Supplementary Fig. S4.3 The best-fit of the model (Equation (2) in Materials and Methods) estimating contribution from plasma virus at different <u>first ATI timepoints to predicting probability of rebound at the second ATI in Cohort 3</u> (supplement to Fig. 4.1B). The x-axis is the sum of viral loads from predictor measurements weighted by per-virion contribution of respective measurements. The horizontal position of black "x" symbols indicates the weighted pre-treatment viral load of different barcodes. Barcodes that did not rebound have y-coordinate = 0, and barcodes that rebounded have y-coordinate =1. The green-shaded bar is the fraction of barcodes (in the given bin) that rebounded. The best-fit parameters and the percent total contribution to the reactivatable pool are in Supplementary Table S4.2.

Supplementary section 4. Prediction of the rebound at 2nd ATI using plasma virus from the first ATI.

		α_3 (Days 313-343)	α_4 (Days 399-416)
**Cohort 3**		0.0569864	0.943014
**ID**	**β**	**Percent total contribution**	
		**Days 313-343**	**Days 399-416**
2_RSk17	0.00195371	3.56669	96.4333
3_RIa17	0.0131013	4.34717	95.6528
5_RFb17	0.0104704	4.27527	95.7247
6_RGj17	0.0166385	2.70506	97.2949
8_34534	0.0198085	1.53049	98.4695
10_RTb17	0.00843898	4.75669	95.2433
11_14D076	0.00804571	10.3572	89.6428
13_RNc17	0.00074842	0.299858	99.7001
15_RYi17	0.000707636	5.00527	94.9947
16_RFa17	0.00547302	5.02208	94.9779
17_RRj17	0.000101355	41.2558	58.7442
18_RBm17	0.0141713	10.2576	89.7424

Supplementary Table S4.2. The best-fit parameters of the model presented in Equation (2) of Materials and Methods, estimating the percent of the total contribution from plasma virus at different timepoints at the first ATI to predicting rebound at the second ATI in Cohort 3. (supplement to Supplementary Fig. S4.1B and Supplementary Fig. S4.3).

Supplementary section 5. Contribution to total cDNA.

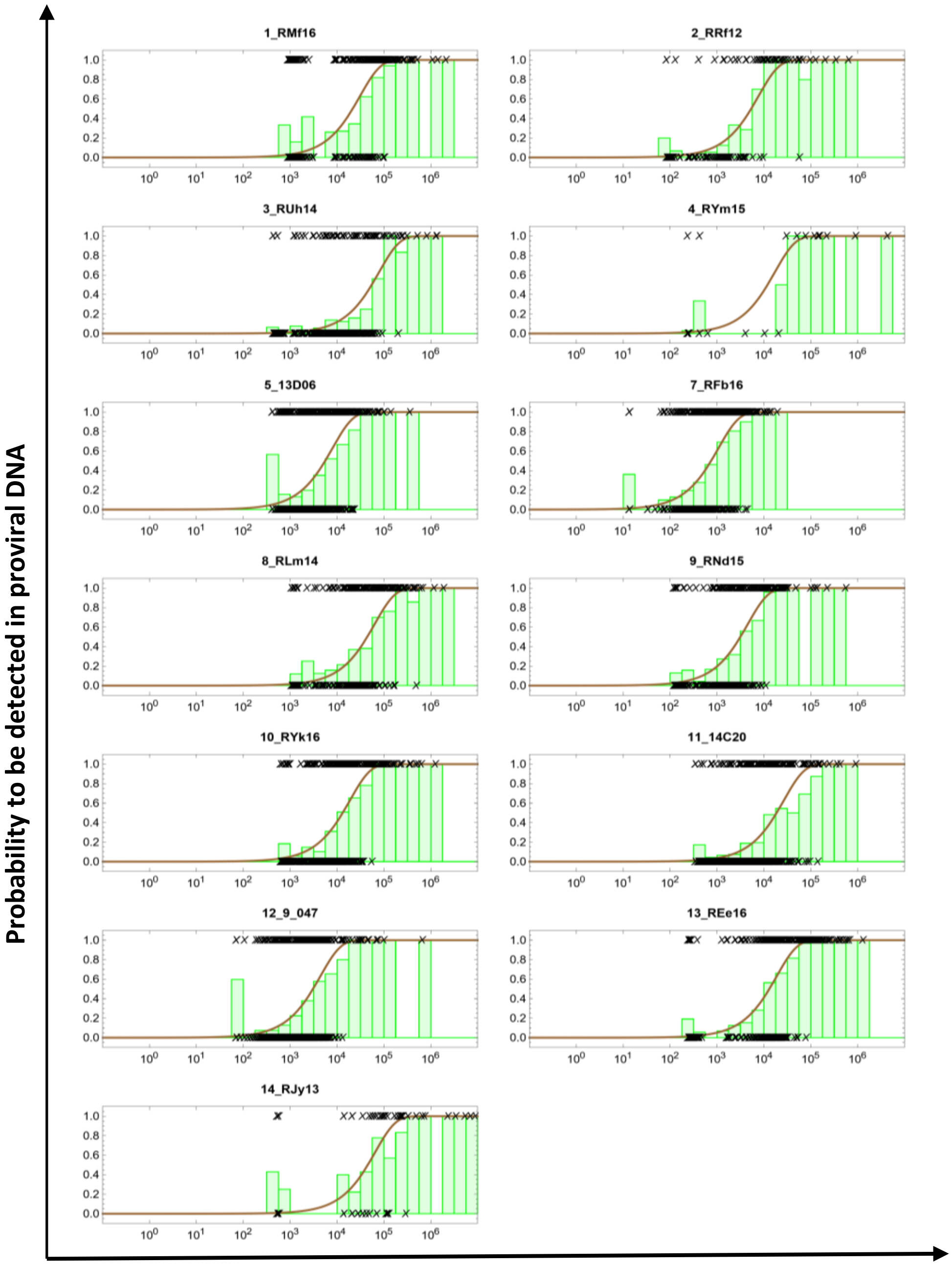

Supplementary Fig. S5.1. The best-fit of the model (Equation (2) in Materials and Methods) estimating contribution from plasma virus at different timepoints at <u>pre-ART to predicting cell-associated DNA during the first round of ART in Cohort 1</u> (supplement to Fig. 5A). The x-axis is the weighted sum of pre-treatment viral loads from different measurements that contributed to the latent reservoir. The horizontal position of black "x" symbols indicates the weighted pre-treatment viral load of different barcodes. Barcodes that were not detected have y-coordinate = 0, and barcodes that were detected in the DNA have y-coordinate =1. The green-shaded bar is the fraction of barcodes (in the given bin) that were detected. The best-fit parameters and the percent total contribution to the proviral DNA are in Supplementary Table S5.1.

Supplementary section 5. Contribution to total cDNA.

	α_1 (Day 7)	α_2(Day 14)
**Cohort 1**	0.709754	0.290246

ID	β	Percent total contribution	
		**Day 7**	**Day 14**
1_RMf16	0.0000320148	1.9781	98.0219
2_RRf12	0.000128904	0.565331	99.4347
3_RUh14	0.0000125632	1.6207	98.3793
4_RYm15	0.000057748	0.0442297	99.9558
5_13D06	0.000123729	17.5787	82.4213
7_RFb16	0.00092126	65.3794	34.6206
8_RLm14	0.0000156671	1.81885	98.1812
9_RNd15	0.000218771	2.80879	97.1912
10_RYk16	0.0000524288	2.73524	97.2648
11_14C20	0.0000370453	6.99703	93.003
12_9_047	0.000224984	24.3354	75.6646
13_REe16	0.0000528708	0.933452	99.0665
14_RJy13	0.0000152774	0.0527404	99.9473

Supplementary Table S5.1. The best-fit parameters of the model presented in Equation (2) of Materials and Methods, estimating the percent of the total contribution from plasma virus at different timepoints pre-ART to predicting cell-associated DNA at the first ART in Cohort 1 (supplement to Fig. 5A and Supplementary Fig. S5.1).

Supplementary section 5. Contribution to total cDNA.

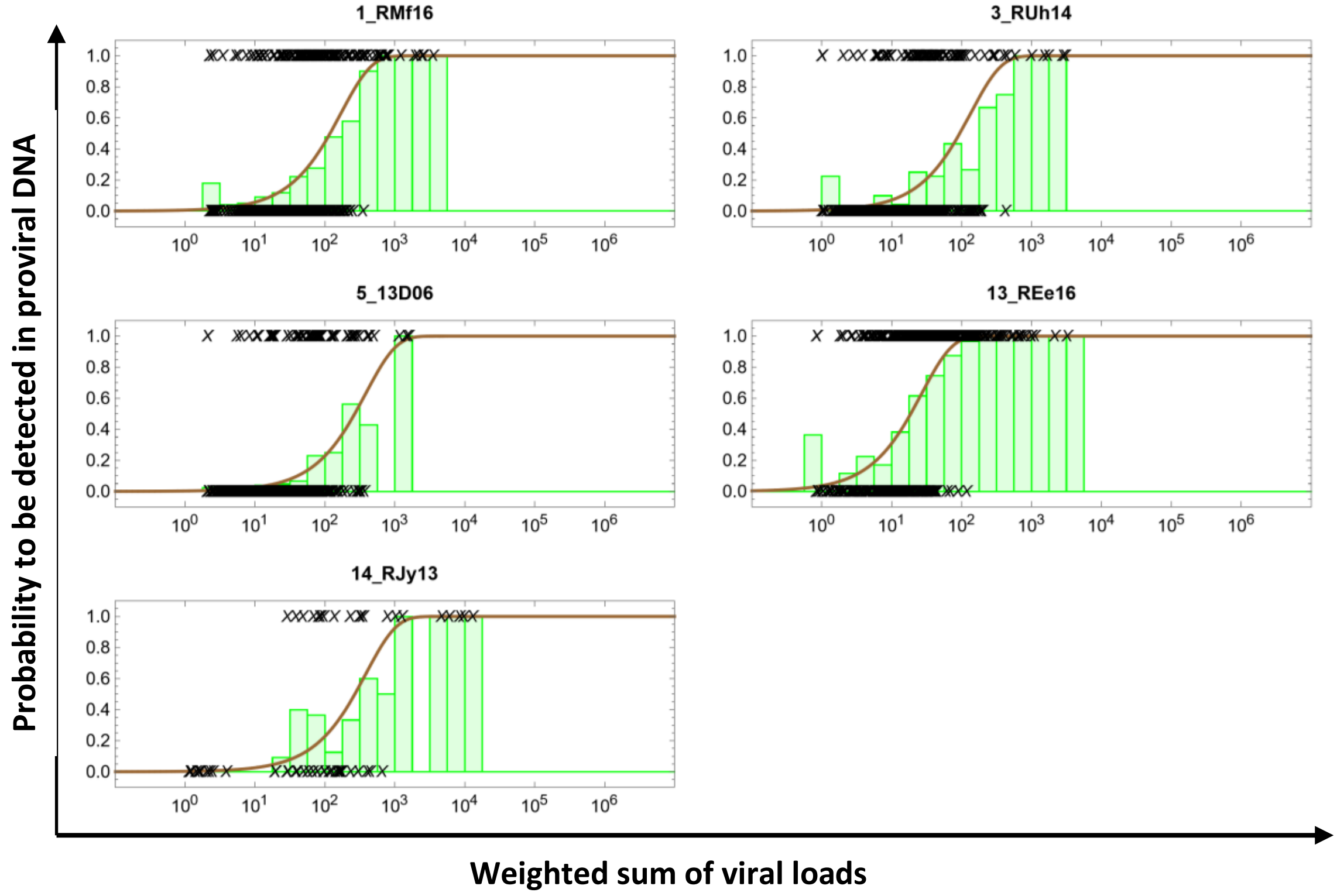

Supplementary Fig. S5.2. The best-fit of the model (Equation (2) in Materials and Methods) estimating contribution from plasma virus at different timepoints at pre-ART to predicting cell-associated DNA at the second round of ART in Cohort 1 (supplement to Fig. 5B). The x-axis is the weighted sum of pre-treatment viral loads from different measurements that contributed to the latent reservoir. The horizontal position of black “x” symbols indicates the weighted pre-treatment viral load of different barcodes. Barcodes that were not detected have y-coordinate = 0, and barcodes that were detected in the DNA have y-coordinate =1. The green-shaded bar is the fraction of barcodes (in the given bin) that were detected. The best-fit parameters and the percent total contribution to the proviral DNA are in Supplementary Table S5.2.

Supplementary section 5. Contribution to total cDNA.

		α_1 (Day 7)	α_2 (Day 14)	α_3 (Days 233-239)	α_4 (Days 281-282)
**Cohort 1**		0.0172497	0.000395477	0.0116878	0.970667
**ID**	**β**	**Percent total contribution**			
		**Day 7**	**Day 14**	**Days 233-239**	**Days 281-282**
1_RMf16	0.00585621	22.4843	62.4648	0.730526	14.3204
3_RUh14	0.00736422	18.5271	63.0509	0.381013	18.041
5_13D06	0.00259525	70.2654	18.4703	1.48164	9.7826
13_REe16	0.0369619	12.1458	72.2675	0.713229	14.8735
14_RJy13	0.0025861	0.826928	87.8571	0.628626	10.6873

Supplementary Table S5.2. The best-fit parameters of the model presented in Equation (2) of Materials and Methods, estimating the percent of the total contribution from plasma virus at different timepoints pre-ART to predicting cell-associated DNA at the second ART in Cohort 1 (supplement to Fig. 5B and Supplementary Fig. S5.2).

Supplementary section 5. Contribution to total cDNA.

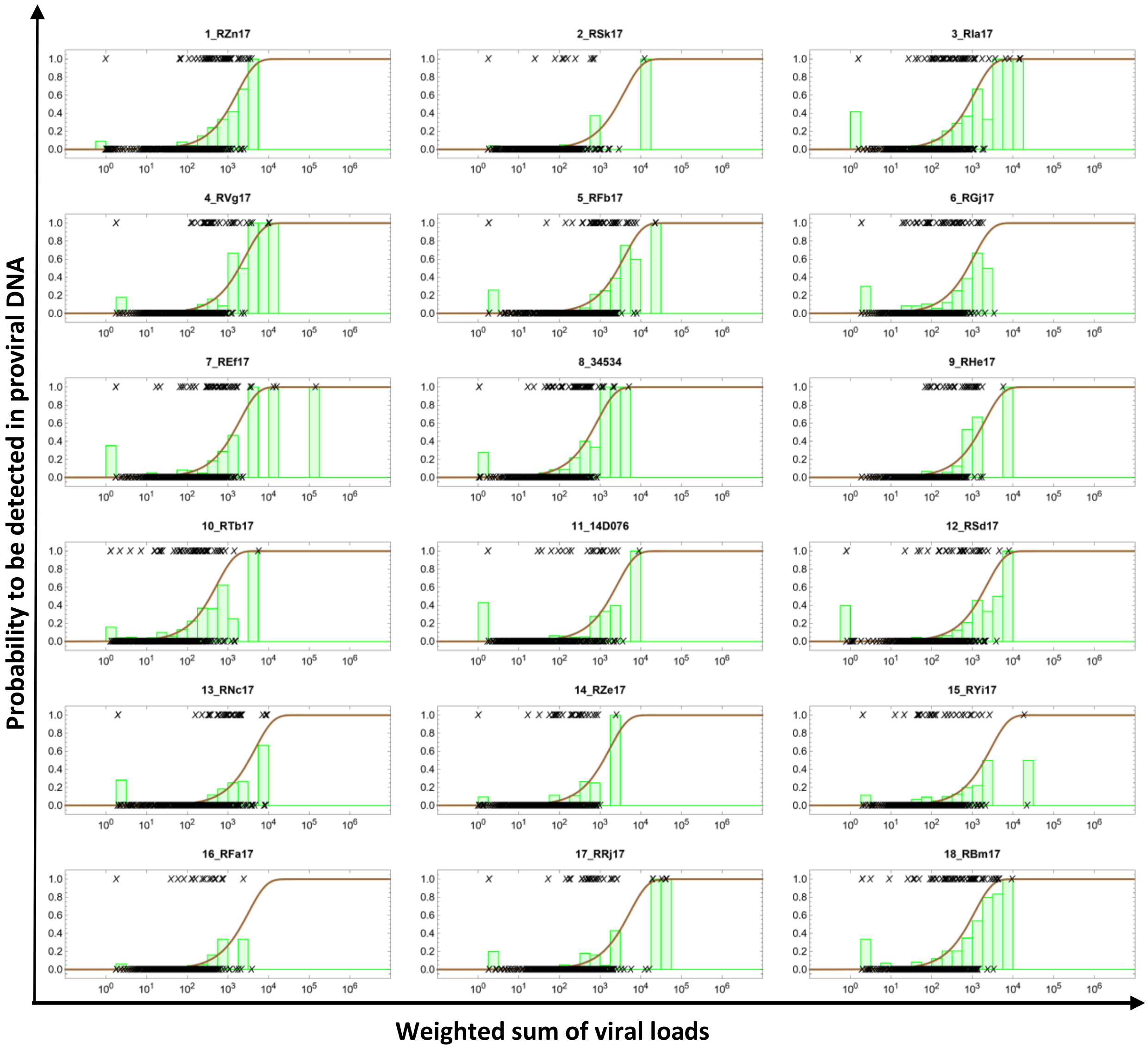

Supplementary Fig. S5.3. The best-fit of the model (Equation (2) in Materials and Methods) estimating contribution from plasma virus at different timepoints at pre-ART to predicting cell-associated DNA at the first round of ART in Cohort 3 (supplement to Fig. 5C). The x-axis is the weighted sum of pre-treatment viral loads from different measurements that contributed to the latent reservoir. The horizontal position of black "x" symbols indicates the weighted pre-treatment viral load of different barcodes. Barcodes that were not detected have y-coordinate = 0, and barcodes that were detected in the DNA have y-coordinate =1. The green-shaded bar is the fraction of barcodes (in the given bin) that were detected. The best-fit parameters and the percent total contribution to the proviral DNA are in Supplementary Table S5.3.

Supplementary section 5. Contribution to total cDNA.

	α_1 (Days 14,15)	α_2 (Days 119-121)
**Cohort 3**	0.0418124	0.958188

ID	β	Percent total contribution	
		**Days 14,15**	**Days 119-121**
1_RZn17	0.000574156	97.9999	2.00015
2_RSk17	0.000254248	69.1412	30.8588
3_RIa17	0.000856255	61.0203	38.9797
4_RVg17	0.00037082	79.599	20.401
5_RFb17	0.000248822	71.7884	28.2116
6_RGj17	0.000907146	95.6315	4.36848
7_REf17	0.000503988	35.3332	64.6668
8_34534	0.00113837	89.3928	10.6072
9_RHe17	0.000481715	98.817	1.18297
10_RTb17	0.00183973	81.3991	18.6009
11_14D076	0.000375117	84.8592	15.1408
12_RSd17	0.000431089	99.1054	0.894622
13_RNc17	0.000209862	81.4823	18.5177
14_RZe17	0.000573579	96.9137	3.08627
15_RYi17	0.00034856	57.0025	42.9975
16_RFa17	0.000309095	84.8801	15.1199
17_RRj17	0.000184814	45.2444	54.7556
18_RBm17	0.000879257	73.2354	26.7646

Supplementary Table S5.3 The best-fit parameters of the model presented in Equation (2) of Materials and Methods, estimating the percent of the total contribution from plasma virus at different timepoints pre-ART to predicting cell-associated DNA at the first ART in Cohort 3 (supplement to Fig. 5C and Supplementary Fig. S5.3).

Supplementary section 5. Contribution to total cDNA.

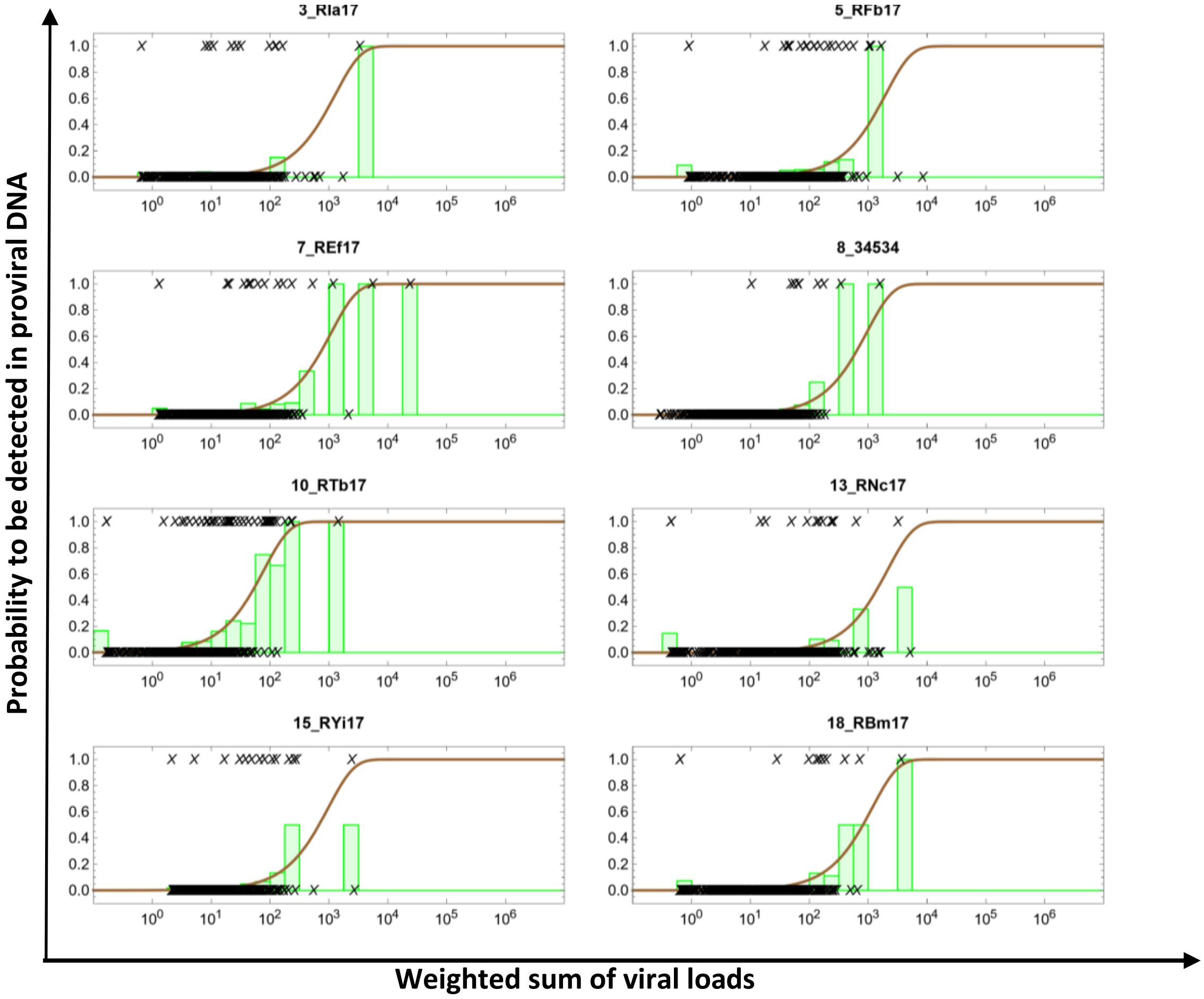

Supplementary Fig. S5.4. The best-fit of the model (Equation (2) in Materials and Methods) estimating contribution from plasma virus at different timepoints at pre-ART to predicting cell-associated DNA at the second round of ART in Cohort 3 (supplement to Supplementary Fig. S5D). The x-axis is the weighted sum of pre-treatment viral loads from different measurements that contributed to the latent reservoir. The horizontal position of black "x" symbols indicates the weighted pre-treatment viral load of different barcodes. Barcodes that were not detected have y-coordinate = 0, and barcodes that were detected in the DNA have y-coordinate =1. The green-shaded bar is the fraction of barcodes (in the given bin) that were detected. The best-fit parameters and the percent total contribution to the proviral DNA are in Supplementary Table S5.4.

Supplementary section 5. Contribution to total cDNA.

	α_1 (Days 14, 15)	α_2 (Days 119-121)	α_3 (Days 313-343)	α_4 (Days 399-416)
**Cohort 3**	0.00645059	0.0335707	0.0185623	0.941416

ID	β	Percent total contribution			
		Day 14, 15	Day 119-121	Days 313-343	Days 399-416
3_RIa17	0.000776784	64.7024	9.38642	0.378618	25.5326
5_RFb17	0.000503175	67.3496	6.01069	0.382634	26.257
7_REf17	0.000901975	31.6289	13.1462	0.0975371	55.1274
8_34534	0.00104858	84.1837	2.26852	0.0683596	13.4795
10_RTb17	0.012655	77.0744	3.99983	0.30346	18.6223
13_RNc17	0.000466382	67.9013	3.50443	0.0280331	28.5662
15_RYi17	0.00102004	62.0025	10.6212	0.462699	26.9136
18_RBm17	0.000848195	72.0187	5.97724	0.79113	21.2129

Supplementary Table S5.4. The best-fit parameters of the model presented in Equation (2) of Materials and Methods, estimating the percent of the total contribution from plasma virus at different timepoints pre-ART to predicting cell-associated DNA at the second ART in Cohort 3 (supplement to Fig. 5D and Supplementary Fig. S5.4).

Supplementary section 6. Prediction of rebound by cDNA vs Plasma.

Comparison number	Cohort	Model	AIC	Log-likelihood	Num. of subjects	Result
**Predicting rebound at first ATI using plasma measurements at Pre-ART or cDNA at first ART**						
1	C1	cDNA in PBMC	3302.91	-1640.45	11	
		Plasma	3015.65	-1496.83		fits better
2	C1	cDNA in LN	3468.76	-1724.38	10	
		Plasma	2923.34	-1450.67		fits better
3	C3	cDNA in PBMC	2249.07	-1106.54	18	
		Plasma	1601.8	-781.898		fits better
**Predicting rebound at the second ATI using plasma measurements at Pre-ART and the first ATI 1 or cDNA at the second ART**						
4	C1	cDNA in PBMC	547.035	-268.517	5	
		Plasma	345.241	-164.62		fits better
5	C1	cDNA in LN	455.949	-223.975	4	
		Plasma	345.241	-164.62		fits better
6	C3	cDNA in PBMC	602.21	-292.105	9	
		Plasma	324.998	-150.499		fits better

Supplementary Table S6.1. Comparisons of the goodness of fit of the models that predict rebound using barcode structure in plasma virus or in cDNA. Models (Equation (2) in the main text) that use plasma virus have consistently better predictive capabilities (by AIC) than ones that use cDNA before the rebound.

Supplementary section 7. Estimating the turnover of the latent reservoir.

Modelling the decay of proviral DNA

Several more recent studies have also estimated the rate of decay of the reservoir during the untreated infection by fitting the exponential decay curve to a fraction of sequences integrated at different times before ART treatment (3-5). Using a similar approach, we can estimate the rate of decay of the reservoir by fitting an exponential decay model to the normalized contribution of plasma virus to both rebound and proviral DNA observed later in infection.

$$L_{ij}(t) = e^{-r_{ij}t} \tag{7.1}$$

Where L_{ij} is the latent reservoir normalized to the last measurement before ART initiation, i can take values "Cohort 1 and 2" or "Cohort 3", j – RIVR or Total DNA; r_{ij} is the decay rate for the corresponding cohort and type of proviral DNA, and t – is the time of exposure to untreated infection between the last measurement before ART and the previous ones. We assume that the decay rate for RIVR is proportional to total proviral DNA by the same value in all cohorts.

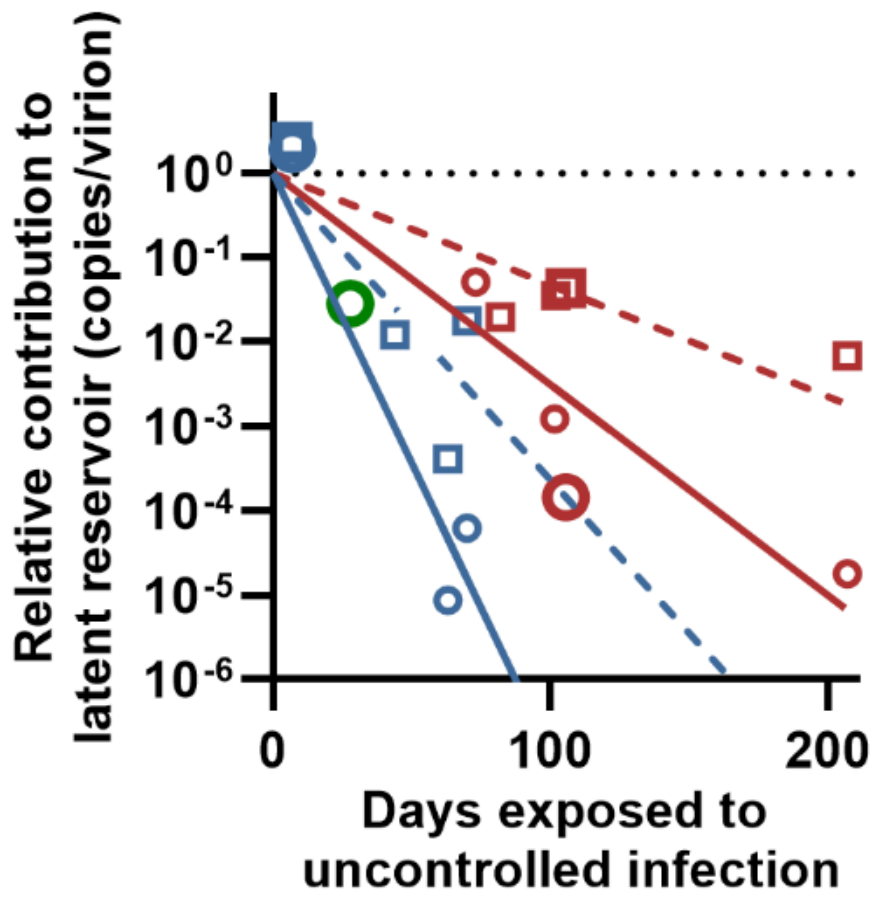

Cohort#	ATI#	Rebound or DNA	Data marker	Function marker
1	1	Rebound	○	——
1	2	Rebound	∘	
2	1	Rebound	○	
1	1	DNA	□	- - -
1	2	DNA	▫	
3	1	Rebound	○	——
3	2	Rebound	∘	
3	1	DNA	□	- - -
3	2	DNA	▫	

Supplementary Fig. S7.1. Estimating turnover of the virus-infected cells during untreated infection.
We fitted the normalized contribution of plasma virus to rebound and proviral DNA at different times to estimate the turnover of the relevant reservoirs, assuming an exponential turnover rate. Individual points indicate the normalized contributions obtained from fitting model (2), with lines indicating the best-fit turnover model (equation 7.1). Data is shown for two treatment interruptions and for proviral DNA in cohorts 1 (blue) and 3 (red). For cohort 2, only data on contributions to the first rebound were available (green). The two SIV infection cohorts (cohorts 1 and 2) show higher turnover than the SHIV cohort. The rebound-initiating proviral DNA decays 1.9 times faster than the total proviral DNA for both groups.

Fitting the normalized contribution of sequences sampled at different times and considering the duration of untreated viremia prior to ART, we estimate that the rebound-initiating population of virus-infected cells has halflife of 4.3 days (95% CI= 3.5 - 5.6) and 12.0 days (95% CI = 9.7 - 15.3) for

Supplementary section 7. Estimating the turnover of the latent reservoir.

cohorts 1 and 3, respectively, during active infection. By contrast, the total proviral DNA reservoir is more stable, with an estimated halflife of 8.3 days (95% CI = 6.2 - 12.6) and 22.7 days (95% CI = 16.8 - 35.1) for cohorts 1 and 3, respectively. These estimated turnover rates are quite different between the cohorts, likely reflective of the different viruses used (SIV vs SHIV), and the lower virulence and infectivity of the latter virus stock. We should note that the 0-values of relative contribution were removed from fitting on the log scale. The fitting with 0-values replaced with the second minimal value encountered in the data is presented in Supplementary Table S7.1, which gives us a similar result.

Model (7.1) was fitted to log-transformed data of normalized contribution, with the values equal to 0 (cohort 2, day 7 and Cohort 1, day 233-239) on the linear scale removed from fitting (method (i)). Here, we demonstrate the robustness of the fitting results by assuming that the 0-values are equal to 8.75×10^{-6} (method (ii)). Ie: we assign zero values to the lowest minimal value of normalized contribution above 0 that was estimated by modelling. The comparisons of the results from both methods are in the Supplementary Table S7.1.

	Method (i), 0 - removed.	Method (ii), 0 - replaced.
Total cell-associated DNA, cohorts 1 and 2.	8.27, CI(6.16, 12.57)	8.16, CI(5.09, 20.58)
Total cell-associated DNA, Cohorts 3.	22.74, CI(16.82, 35.06)	23.05, CI(14.21, 60.00)
The ratio of halflife between the total DNA and the rebound initiating	1.92, CI(1.23, 2.60)	2.10, CI(0.79, 3.41)

Supplementary Table S7.1. Comparison of best-fit parameters of the model (3) depending on whether we remove values equal to 0 in the data or replace them with the next smallest value.

Supplementary section 8. Timing of seeding of HIV reservoir

Article	Duration of HIV infection prior to ART (years)	Considered pre-ART time interval (years before ART initiation)	Number of patients	% of total sequences integrated at the pre-ART interval	Comments
Abrahams et al. 2019 (7)	2.7-6.9	0 - 1	9	71	Inducible DNA.
Joseph et al. 2024 (8)	2.6-7.3	0 - 1	18	69	Inducible DNA.
				60	Total proviral DNA.
Brodin et al. 2016 (5)	5.6-16.1	0 - 1	10	60	Total proviral DNA.
Kankaka et al. 2023 (9)	2.6-11.5	0 - 1/3 of the duration of the untreated infection	11	48.7	Inducible DNA. (Rakai LVR cohort)
	2.7-6.9	0 - 1/3 of the duration of the untreated infection	9	57.9	Inducible DNA. (CAPRISA Cohort)
Brooks et al. 2020 (4)	2.75 - 6.25	0 - 4.2	13	56.8	Total proviral DNA.
Pankau et al. 2020 (3)	5.4–18	At ART initiation	6	49.7	Total proviral DNA.
Kinloch et al. 2023 (10)	2.2-26.5	0 - 1	6	40	Total proviral DNA.
			2	89	Inducible DNA.
			1	80	Rebound-initiating virus
Shahid et al. 2024 (11)	1.9-12	0 - 1	6	41.8	Total proviral DNA
			1	75	Rebound-initiating virus

Supplementary Table S8.1. Timing of the contribution of the plasma virus before ART to the latent reservoir in the HIV studies. Despite the studies having different numbers of samples and different durations of uncontrolled infection, most of the proviral DNA is integrated at time intervals immediately prior to ART initiation; the outgrowth virus and rebounding virus have even more pronounced bias towards treatment time. The data is visualized in Fig. 7 in the main text.

The method of acquisition of published values that are presented in Supplementary Table S8.1.

Abrahams et al. 2019. All parameters were taken from the text (page 4) and Table 1.

Joseph et al. 2024. All parameters were taken from the text (page 3) and Table 1.

Supplementary section 8. Timing of seeding of HIV reservoir

Brodin et al. 2016. All parameters were taken from the text (page 5) and Table 1. The range of the duration of HIV infection prior to ART was estimated at approximately, taking the largest and smallest number of years passed from the estimated date of infection to the last sample before ART initiation. According to Fig. 1, the last samples were taken almost at the time of ART except for subject p2, which was excluded from the calculation of minimal time.

Kankaka et al. 2023. The percentage of integrated DNA was taken from Supplementary Table 1 for the Rakai LVR cohort and extracted with WebPlotDigitazer 4.2 from Fig. 3 for the CAPRISA cohort. The values reported in Supplementary Table S8.1 are averages among all patients and all genes. The durations of exposure to uncontrolled infection were taken from Fig. 3 for both cohorts.

Brooks et al. 2020. The percentage of integrated DNA is the value presented in Fig. 4.C. The durations of uncontrolled infection are from Table 1. Time to the last sample before ART was estimated as the difference of time exposure to uncontrolled infection (EDI to ART Start) in Table 1 and the estimated date of infection (EDI) to the last ART-naïve sample in S1 Table.

Pankau et al. 2020. The percentage of integrated DNA was extracted with WebPlot Digitiser 4.2 from the last bar of a bar plot in Fig. 4B for the gag gene and S5 Fig. B. for the env gene. The duration of exposure to the untreated infection is from the text (page 3). The timing of the last ART-naïve measurement is taken from Fig. 1 and is approximately equal to the time of ART initiation.

Kinloch et al. 2023. The percentage of integrated DNA was calculated using the data provided by the authors of the manuscript. The bin size of 1 year before ART initiation was chosen to be consistent with most studies in Supplementary Table S8.1. The durations of uncontrolled infection are from Table 1. The integration dates after the ART initiation are assumed to be the estimation errors and counted in the last bin before ATI initiation. The dates of ART initiation were taken from article (10), except for patient BC-004, for whom the date of ART initiation was not mentioned in the text and was extracted from Fig. 9. The low-level viremia on ART was not counted as a rebounding virus since the drug-resistant mutations may bias the estimated integration dates of the rebounding reservoir. Only the rebound during ART interruption in patient BC-001 is counted as a rebounding virus.

Shahid et al. 2024. The percentage of integrated DNA was calculated using the data provided by the authors of the manuscript. The bin size of 1 year before ART initiation was chosen to be consistent with most studies in Supplementary Table S8.1. The durations of uncontrolled infection are from Table 1. The integration dates after the ART initiation are assumed to be the estimation errors and

counted in the last bin before ATI initiation. The dates of ART initiation were taken from the article (11) text, except for the date of the second ART initiation for participant 1, which was extracted from Figure 10. The viremia blip on ART in participant 5 was not counted as a rebounding virus since the drug-resistant mutations may bias the estimated integration dates of the rebounding reservoir. Only the rebound during ART interruption in participant 1 is counted as a rebounding virus. The integration dates of rebounding viruses that fell to the treatment interruption time were excluded from analysis.

Supplementary section 9. Fitting the control subjects only.

	Cohort #	Predicting rebound or cDNA	Predicting	Predictors	Number of subjects	Mean total contribution at each time point			
						Time Point 1	Time Point 2	Time Point 3	Time Point 4
**Control and treated subjects**	**1**	rebound	ATI #1	Pre-treatment	13	8.421 (16.640)	91.579 (16.640)		
	**2**	rebound	ATI #1	Pre-treatment	6	0	35.387 (14.290)	64.613 (14.290)	
	**3**	rebound	ATI #1	Pre-treatment	18	5.162 (7.891)	94.838 (7.891)		
	**1**	rebound	ATI #2	Pre-treatment +ATI #1	7	0.681 (0.816)	8.560 (4.304)	0	90.759 (4.057)
	**3**	rebound	ATI #2	Pre-treatment +ATI #1	12	1.157 (0.823)	0.851 (0.303)	6.729 (9.859)	91.263 (9.599)
	**1**	rebound	ATI #2	ATI #1	7			0.259 (0.159)	99.742 (0.159)
	**3**	rebound	ATI #2	ATI #1	12			7.782 (10.956)	92.218 (10.956)
	**1**	cDNA	cDNA #1	Pre-treatment	14	9.758 (18.288)	90.243 (18.288)		
	**3**	cDNA	cDNA #1	Pre-treatment	18	77.936 (18.724)	22.064 (18.724)		
	**1**	cDNA	cDNA #2	Pre-treatment +ATI #1	5	24.850 (26.677)	60.822 (25.803)	0.787 (0.413)	13.541 (3.350)
	**3**	cDNA	cDNA #2	Pre-treatment +ATI #3	8	65.858 (15.556)	6.864 (3.819)	0.314 (0.253)	26.964 (12.437)
**Control subjects**	**1**	rebound	ATI #1	Pre-treatment	6	6.208 (9.318)	93.792 (9.318)		
	**2**	rebound	ATI #1	Pre-treatment	3	6.94E-09 (5.28E-09)	38.585 (19.115)	61.415 (19.115)	
	**3**	rebound	ATI #1	Pre-treatment	3	4.732 (6.676)	95.268 (6.676)		
	**1**	rebound	ATI #2	Pre-treatment +ATI #1	3	0	9.252 (2.154)	9.44E-06 (1.95E-06)	90.748 (2.154)
	**3**	rebound	ATI #2	Pre-treatment +ATI #1	9	1.158 (0.895)	3.276 (1.448)	1.406 (0.511)	94.160 (1.281)
	**1**	rebound	ATI #2	ATI #1	9			0.531 (0.114)	99.469 (0.114)
	**3**	rebound	ATI #2	ATI #1	3			3.394 (1.222)	96.606 (1.222)
	**1**	cDNA	cDNA #1	Pre-treatment	3	10.167 (13.993)	89.833 (13.993)		
	**3**	cDNA	cDNA #1	Pre-treatment	5	66.360 (26.689)	33.640 (26.689)		
	**1**	cDNA	cDNA #2	Pre-treatment +ATI #1	5	11.111 (13.496)	74.106 (15.806)	1.61E-05 (2.01E-07)	14.783 (2.310)
	**3**	cDNA	cDNA #2	Pre-treatment +ATI #3	3	70.143 (20.271)	7.705 (5.229)	4.42E-12 (3.20E-12)	22.152 (15.272)

Supplementary Table S9.1 Comparison of mean percent of total contribution estimates when we fit the Equation (2) from Materials and Methods to both treated and control subjects and control subjects only. Numbers in brackets are standard deviations. cDNA stands for cell-associated DNA. The first and the second rounds of ATIs are denoted as ATI #1 and ATI #2. Fitting without subjects that underwent different interventions gives similar results.

1. S. S. Docken *et al.*, Preferential selection of viral escape mutants by CD8+ T cell 'sieving' of SIV reactivation from latency. *PLoS Pathog.* **19**, e1011755 (2023).
2. E. G. Viox *et al.* (2022) Impact of CD4 binding site bNAbs on barcoded TF-SHIV-D rebound in macaques at ATI. in *Conference on Retroviruses and Opportunistic Infections* (Virtual).
3. M. D. Pankau *et al.*, Dynamics of HIV DNA reservoir seeding in a cohort of superinfected Kenyan women. *PLoS Pathog.* **16**, e1008286 (2020).
4. K. Brooks *et al.*, HIV-1 variants are archived throughout infection and persist in the reservoir. *PLoS Pathog.* **16**, e1008378 (2020).
5. J. Brodin *et al.*, Establishment and stability of the latent HIV-1 DNA reservoir. *Elife* **5** (2016).
6. M. Pinkevych *et al.*, Predictors of SIV recrudescence following antiretroviral treatment interruption. *Elife* **8**, e49022 (2019).
7. M. R. Abrahams *et al.*, The replication-competent HIV-1 latent reservoir is primarily established near the time of therapy initiation. *Sci. Transl. Med.* **11** (2019).
8. S. B. Joseph *et al.*, The timing of HIV-1 infection of cells that persist on therapy is not strongly influenced by replication competency or cellular tropism of the provirus. *PLoS Pathog.* **20**, e1011974 (2024).
9. E. N. Kankaka *et al.*, Dating reservoir formation in virologically suppressed people living with HIV-1 in Rakai, Uganda. *Virus Evol* **9**, vead046 (2023).
10. N. N. Kinloch *et al.*, HIV reservoirs are dominated by genetically younger and clonally enriched proviruses. *mBio* **14**, e02417-02423 (2023).
11. A. Shahid *et al.*, The replication-competent HIV reservoir is a genetically restricted, younger subset of the overall pool of HIV proviruses persisting during therapy, which is highly genetically stable over time. *J. Virol.* **98**, e01655-01623 (2024).
